# Bayesian Hodges-Lehmann tests for statistical equivalence in the two-sample setting: Power analysis, type I error rates and equivalence boundary selection in biomedical research

**DOI:** 10.1186/s12874-021-01341-7

**Published:** 2021-08-17

**Authors:** Riko Kelter

**Affiliations:** Department of Mathematics, University of Siegen, Walter-Flex-Str. 3, Siegen, Germany

**Keywords:** Bayesian equivalence testing, Bayesian testing, Student’s t-test, Bayesian Biostatistics, Bayes factor, Region of practical equivalence (ROPE)

## Abstract

**Background:**

Null hypothesis significance testing (NHST) is among the most frequently employed methods in the biomedical sciences. However, the problems of NHST and *p*-values have been discussed widely and various Bayesian alternatives have been proposed. Some proposals focus on equivalence testing, which aims at testing an interval hypothesis instead of a precise hypothesis. An interval hypothesis includes a small range of parameter values instead of a single null value and the idea goes back to Hodges and Lehmann. As researchers can always expect to observe some (although often negligibly small) effect size, interval hypotheses are more realistic for biomedical research. However, the selection of an equivalence region (the interval boundaries) often seems arbitrary and several Bayesian approaches to equivalence testing coexist.

**Methods:**

A new proposal is made how to determine the equivalence region for Bayesian equivalence tests based on objective criteria like type I error rate and power. Existing approaches to Bayesian equivalence testing in the two-sample setting are discussed with a focus on the Bayes factor and the region of practical equivalence (ROPE). A simulation study derives the necessary results to make use of the new method in the two-sample setting, which is among the most frequently carried out procedures in biomedical research.

**Results:**

Bayesian Hodges-Lehmann tests for statistical equivalence differ in their sensitivity to the prior modeling, power, and the associated type I error rates. The relationship between type I error rates, power and sample sizes for existing Bayesian equivalence tests is identified in the two-sample setting. Results allow to determine the equivalence region based on the new method by incorporating such objective criteria. Importantly, results show that not only can prior selection influence the type I error rate and power, but the relationship is even reverse for the Bayes factor and ROPE based equivalence tests.

**Conclusion:**

Based on the results, researchers can select between the existing Bayesian Hodges-Lehmann tests for statistical equivalence and determine the equivalence region based on objective criteria, thus improving the reproducibility of biomedical research.

## Background

Hypothesis testing is among the most widely established statistical methods in the biomedical sciences [1, 2]. The often inadequate use of null hypothesis significance testing (NHST) has been debated widely [3, 4], and the consequences pose severe problems for scientific progress. Among the problems of NHST are inflated type I error rates [5, 6], the inability to make use of optional stopping [7–9] and problems with the interpretation of censored data [7, 8] which are frequently observed in the biomedical sciences, for example in clinical trials. Those problems are caused mostly by the fact that frequentist NHST and *p*-values violate the likelihood principle [10], which is of paramount importance in statistical science. In contrast, Bayesian inference is following the likelihood principle [8, 11, 12] and most of the above problems disappear when utilising Bayesian data analysis and, in particular, Bayesian hypothesis tests [13].

First, among the advantages of Bayesian inference is the easier interpretation of interval estimates like Bayesian credible or highest-posterior-density (HPD) intervals compared to frequentist confidence intervals [14]. The former quantify the probability that the parameter is located in a specific range of values given the data, while the latter quantify the probability that the parameter is covered by the interval under hypothetical repetition of the study^1^. Also, in Bayesian inference, probabilistic statements about parameters can be made instead of relying only on likelihood-based reasoning [13, 16].

Second, Bayesian tests follow the likelihood principle (LP) [10] which itself implies the stopping rule principle (SRP) and the censoring principle (CP), see Berger and Wolpert [8]. The SRP states that is does not matter whether a study is designed with fixed sample size or until time or funding runs out: The results of a hypothesis test should not be influenced by the stopping rule, that is, the decision when to stop sampling. In sharp contrast, frequentist tests conflict with the LP and thus also with the SRP and will yield different results depending on which intentions researchers had: “The irrelevance of stopping rules... restores a simplicity and freedom to experimental design that had been lost by emphasis on significance levels (in the sense of Neyman and Pearson).... The irrelevance of stopping rules is one respect in which Bayesian procedures are more objective than classical ones. Classical procedures insist (...) that the intentions of the experimenter are crucial to the interpretation of data, that 20 successes in 100 observations means something quite different if the experimenter intended the 20 successes than if he intended the 100 observations.”Edwards et al. ([7], p. 239)

That is, the results of a frequentist test can be significant or not depending on whether one started with the intention of a fixed or variable sample size even when the *same* data are observed in both cases. Bayesian tests do not suffer from this situation [17, 18]. This is of huge importance for practical research, as optional stopping allows researchers to stop recruiting study participants and report the results of the analysis when the data show overwhelming evidence after only a fraction of the originally planned sample size is recruited. The consequences are substantial for the biomedical sciences because the ethical obligations for study participants are profound. Additionally, the possibility to make use of optional stopping prevents waste of research resources [19]. As noticed by Berger and Wolpert, the *“theoretical and practical implications of the SRP to such fields as sequential analysis and clinical trials are enormous.”* ([8], p. 74).

Third, the interpretation of censored data is simplified in the Bayesian approach: The censoring principle (CP) is another consequence of the LP and implies that the interpretation of data which could have been censored but were not is identical to the interpretation of data where no censoring was possible at all. This is also of huge value in biomedical research, compare Pratt [20] and Dawid [21]. Again, Bayesian inference is in accordance with the CP, so Bayesian hypothesis testing is simplified compared to frequentist hypothesis testing when data are censored [22].

Due to the advantages of Bayesian inference, recent years have brought the advent of various Bayesian hypothesis tests which were invented to replace or complement frequentist null hypothesis significance tests and *p*-values.

For example, in randomised controlled trials (RCT), the two-sample Student’s and Welch’s *t*-test are among the most frequently carried out hypothesis tests [23, 24]. Often, the goal is to test the efficacy of a new treatment or medication and study the size of the effect between two groups. Usual research designs recruit a treatment and control group and measure the differences in a response variable between them. The status quo in medical research for judging if a new medication is more effective than the existing one is the *p*-value, which states whether the researcher can interpret the observed difference as significant, that means unlikely to have occurred under the assumption of the null hypothesis. The dominance of *p*-values when comparing two groups in the biomedical sciences is overwhelming: Nuijten et al. [23] reported an extensive meta-analysis, which showed that out of 258105 *p*-values which were reported in journals between 1985 and 2013, 26% corresponded to a *t*-statistic, compare also Wetzels et al. [24]. The recently published analysis of the efficacy of hydroxychloroquine in patients with COVID-19 of Chen et al. [25] shows that such two-sample comparisons via NHST and *p*-values remain the gold standard in biomedical research.

## Statistical equivalence testing

The statistical model of the frequentist two-sample Student’s t-test assumes normally distributed data with identical variances $Y_{1i}\sim \mathcal {N}\left (\mu _{1},\sigma ^{2}\right), Y_{2j}\sim \mathcal {N}\left (\mu _{2},\sigma ^{2}\right)$ and sample sizes $i,j=1,...,n, n\in \mathbb {N}$. It tests the null hypothesis of no difference *H*_0_:*μ*_2_=*μ*_1_ against the alternative *H*_1_:*μ*_2_≠*μ*_1_. If the assumption of identical variances in both groups and the assumption of identical sample sizes *i*=*j* is removed, the situation leads to the Behrens-Fisher-problem, to which only approximate solutions exist until today. The typical approach is called *Welch’s two-sample t-test*, and is quite reliable in practice.

Bayesian counterparts to the frequentist two-sample t-test have been developed since 2005. Gönen et al. [26] built on the original proposal of Jeffreys [27]. Rouder et al. [28] extended the solution of Gönen et al. [26], and further modifications were proposed by Wetzels et al. [29], Wang and Liu [30], Gronau et al. [31] and Kelter [32, 33].

However, most of these approaches focus on testing a precise point null hypothesis *H*_0_:*δ*=0, where *δ*=(*μ*_1_−*μ*_2_)/*σ* is the effect size according to Cohen ([34], p. 20). The philosophical problems when using precise point null hypotheses have been debated for a long time in the statistical literature, compare Berger, Brown and Wolpert [35], Rouder et al. [28], Kruschke and Liddell [36], and Lakens et al. [37, 38]. Rouder et al. [28] stressed: “It is reasonable to ask whether hypothesis testing is always necessary. In many ways, hypothesis testing has been employed (...) too often and too hastily (...). To observe structure, it is often sufficient to plot estimates of appropriate quantities along with measures of estimation error (Rouder & Morey, 2005). As a rule of thumb, hypothesis testing should be reserved for those cases in which the researcher will entertain the null as theoretically interesting and plausible, at least *approximately*.”Rouder et al. ([28], p. 235)

In biomedical research, it is necessary to consider alternatives to *precise* hypothesis tests, because it is reasonable to assume *any* kind of effect, although often a negligibly small one. As a consequence, the precise null hypothesis *H*_0_:*δ*=0 is always false. Precise hypothesis tests, whether frequentist or Bayesian, suffer from the property that the null hypothesis *H*_0_:*δ*=0 is always rejected for large enough sample sizes, even for tiny effects *δ*>0 which are scientifically irrelevant.

Concerning the questionable practice of using *precise* hypothesis tests as the standard method in biomedical research, Berger et al. [39] noted: “The decision whether or not to formulate an inference problem as one of testing a precise null hypothesis centers on assessing the plausibility of such an hypothesis. Sometimes this is easy, as in testing for the presence of extrasensory perception, or testing that a proposed law of physics holds. Often it is less clear. In medical testing scenarios, for instance, it is often argued that any treatment will have some effect, even if only a very small effect, and so exact equality of effects (between, say, a treatment and a placebo) will never occur.”Berger, Brown and Wolpert ([35], p. 145)

As exact equality of effects is highly unrealistic in almost all biomedical research settings, it is reasonable to consider *equivalence testing* as a more appropriate alternative instead [32, 36–38, 40]. Equivalence tests replace a null hypothesis *H*_0_:*δ*=0 with *H*_0_:*l*≤*δ*≤*u* for prespecified boundaries *l* and *u*, like *l*=−0.1 and *u*=0.1 [41]. The earliest approaches to what is today called equivalence testing range back to Hodges and Lehmann [42] who considered testing interval hypotheses from a frequentist perspective. Thus, testing for statistical equivalence by means of a Hodges-Lehmann test replaces a precise hypothesis with an interval hypothesis. An overview about frequentist approaches to equivalence testing are given by Lakens et al. [37, 38], but this paper focusses on Bayesian equivalence tests because of the previously outlined advantages of Bayesian statistics in biomedical research. For early approaches of Bayesian equivalence testing see also Lindley [43].

Given the general recommendation of a shift towards the Bayesian paradigm to prevent the problems of NHST and *p*-values, and given the simultaneous recommendation to consider equivalence testing approaches instead of precise hypothesis tests, researchers are faced with several problems when trying to implement such a shift: First, the idea of equivalence testing is appealing, but the formulation of a hypothesis in the approach is complicated. It remains unclear based on which criteria to choose the boundaries *l* and *u* of an imprecise hypothesis like *H*_0_:*l*≤*δ*≤*u*. Second, multiple proposals have been made on how to conduct Bayesian equivalence tests. These include the proposal of the region of practical equivalence (ROPE) of Kruschke [22, 36], which itself can be implemented in three variants. Other approaches favour the Bayes factor [44, 45] and the Bayes factor vs. ROPE index which was proposed by Makowski et al. [46, 47].

The availability of multiple proposals is insofar troubling as even for precise hypotheses (for which the underlying statistical theory is much better developed) it is still debated which evidence measure is appropriate in practice [40, 46]. Some authors argue for the use of the Bayes factor [13, 16, 48], while others regard it as problematic [49]. For example, well-known problems of the Bayes factor include its sensitivity to the prior modeling [50] and the computation of the necessary marginal likelihoods [51]. The latter is often possible only via advanced numerical techniques like the Savage-Dickey density ratio [52–54] or bridge sampling [55, 56].

Bayesian equivalence tests which employ Bayes factors have been proposed by Morey et al. [41] and van Ravenzwaaij et al. [44]. Solutions based on the ROPE have been championed by Kruschke and Liddell [22, 36] and Kelter [32], and a connection between the Bayes factor and the ROPE has been identified by Liao et al. [57].

## Contributions

Now, this paper addresses two connected problems:

1. By now it remains unclear which approach to Bayesian equivalence testing is preferable in practice for a specific statistical method like the two-sample t-test. The decision is complicated by the fact that it remains unknown how the existing approaches behave regarding their type I error rate, their power to detect an existing effect, and their sensitivity to the prior modeling.
2. The selection of the equivalence region (or interval hypothesis) itself presents a major obstacle to use equivalence tests in practice. Although there exists a variety of approaches how to select the equivalence region, none of these is based on objective statistical criteria like the ones mentioned in the previous point.

This paper proposes a new approach to determine the equivalence region in Bayesian equivalence tests in the two-sample setting based on objective criteria like the resulting error rates, power and robustness to prior selection. Therefore, an extensive simulation study is carried out to investigate the first problem. The results are then used to provide a new method to determine the equivalence region for Bayesian equivalence tests, thereby providing a solution to the second problem.

This helps to decide which Bayesian equivalence testing approach should be used in practice for one of the most frequently carried out procedures in the biomedical sciences. Also, it shows how to determine the equivalence region in practice. Via the results the benefits and limitations of the existing approaches are revealed and it is shown how to select the sample size and equivalence region to achieve a desired power and type I error control in contemporary Bayesian equivalence tests. As shown by Makowski et al. [46] and Kelter [33], a careful calibration of Bayesian hypothesis tests regarding the prior hyper-parameters and the sample size is necessary to benefit from a shift towards these Bayesian tests. However, these works were concerned primarily with precise Bayesian hypothesis tests, not with equivalence testing.

Specifically, answers to the following research questions are provided:

- Which type I rates are attained by the various Bayesian equivalence testing approaches? How do these error rates depend on sample size?
- Which sample size is necessary for a selected Bayesian equivalence testing approach to detect a prespecified (e.g. small, medium or large) effect size?
- How robust are the different Bayesian approaches to equivalence testing concerning the prior modeling?
- How does the size of the equivalence region influence the above results? That is, how do type I and II error rates, power and robustness to the prior elicitation vary when the size of the equivalence region is expanded or narrowed?
- How can the equivalence region be determined in practice via the results?

The plan of the paper is as follows: First, the existing approaches to Bayesian equivalence testing are outlined briefly. For readers unfamiliar with traditional frequentist equivalence testing approaches, Appendix A provides a brief overview for comparison^2^. Subsequently, an overview about the existing approaches how to determine the equivalence region is provided. This helps to avoid the claim of arbitrariness against the use of equivalence tests in comparison with precise hypothesis tests. Also, it shows that the proposed method to include objective criteria like type I error rates, power and robustness to the prior selection is new and has several advantages over existing methods. A motivating example illustrates the challenges of using equivalence tests in practice.

Third, the design of the simulation study is detailed. Fourth, the results of the study are presented and discussed. Then, the available approaches to Bayesian equivalence testing are compared and guidance is given when and why to use which approach. The motivating example is revisited to show how to use the results in practice to determine the equivalence region boundaries and attain a desired type I error rate and power. Finally, some challenges in implementing Bayesian equivalence testing and directions for future research are discussed.

## Bayesian approaches to equivalence testing

This section presents the existing Bayesian approaches to equivalence testing. First, the solutions based on the Bayes factor proposed by Morey et al. [41] and van Ravenzwaaij et al. [44] are detailed. Second, the proposals based on the region of practical equivalence (ROPE) made by Kruschke [36, 58], Kruschke and Liddell [22] and Kelter [32] are outlined.

### Bayes factors for equivalence testing

Bayesian hypothesis testing often is associated with the Bayes factor (BF). The Bayes factor *BF*_01_ is a predictive updating factor and measures the change in relative beliefs about both hypotheses *H*_0_ and *H*_1_ under consideration, given the data *x*:

1 $$\begin{array}{*{20}l} \underbrace{\frac{\mathbb{P}(H_{0}|x)}{\mathbb{P}(H_{1}|x)}}_{\text{Posterior odds}} = \underbrace{\frac{f(x|H_{0})}{f(x|H_{1})}}_{BF_{01}(x)} \cdot \underbrace{\frac{\mathbb{P}(H_{0})}{\mathbb{P}(H_{1})}}_{\text{Prior odds}} \end{array} $$

The Bayes factor *BF*_01_ is the ratio of the two marginal likelihoods *f*(*x*|*H*_0_) and *f*(*x*|*H*_1_) of both models, and these are calculated by integrating out the respective model parameters according to their prior distributions. Generally, the calculation of the marginal likelihoods becomes complex for non-trivial models [51, 59], and in high-dimensional settings often numerical techniques are preferred as a consequence [51, 52]. In the Bayesian two-sample t-test, the Bayes factor is used for testing the null hypothesis *H*_0_:*δ*=0 of no effect against the one- or two-sided alternative *H*_1_:*δ*>0,*H*_1_:*δ*<0 or *H*_1_:*δ*≠0, under the assumption of two independent samples and identical standard deviations *σ* in both groups. To translate a given Bayes factor into a statement about the evidence concerning *H*_0_ and *H*_1_, several authors including Jeffreys [60], Kass and Raftery [61], Goodman [62], Lee and Wagenmakers [63], Held and Ott [64] or Van Doorn et al. [65] have offered scales. For example, according to Van Doorn et al. [65], a Bayes factor *BF*_10_≥3 should be interpreted as moderate evidence for the alternative *H*_1_ relative to the null hypothesis *H*_0_, and a Bayes factor *BF*_10_≥10 corresponds to strong evidence for the alternative *H*_1_ relative to *H*_0_.

Among the first proposals for Bayesian equivalence tests which use the Bayes factor was the model of Morey and Rouder [41]. Morey and Rouder [41] separate between three types of hypotheses: The *nil hypothesis*, which states that a parameter or effect is precisely zero, the *null hypothesis*, which may be restricted to a nil hypothesis but may also allow for values which deviate slightly from the nil, and the *default hypothesis*, which refers to a hypothesis which is assumed to be true unless sufficient evidence is presented against it. Morey and Rouder [41] take the default position that the nil hypothesis never holds to arbitrary precision, that is, there is always some kind of effect. As a consequence, they aim to establish a region of parameter values around the nil hypothesis which are not “materially significant”, referring to the early ideas of Hodges and Lehmann [42].

Concerning the two-sample t-test, Morey and Rouder [41] started from the standard Bayesian two-sample t-test model which uses a nil hypothesis:

$$\begin{array}{*{20}l} &H_{0}^{\text{JZS}}:\delta \sim \mathbbm{1}_{\{0\}}\\ &H_{1}^{\text{JZS}}:\delta \sim C(0,1) \end{array} $$

where *C*(0,1) is a Cauchy distribution with scale parameter *γ*=1 under the alternative $H_{1}^{\text {JZS}}$, and *𝟙*_{0}_ is the Dirac measure on 0 with *𝟙*_{0}_(0)=1 and else zero. Choosing Jeffreys’ prior *p*(*σ*^2^)=1/*σ*^2^ for the prior on *σ*^2^ in both groups, this model is known as the Jeffreys-Zellner-Siow (JZS) prior, compare Rouder et al. [28].

### The overlapping hypotheses model

Instead of using the above model which employs a nil hypothesis, Morey and Rouder [41] proposed the following model with overlapping hypotheses:

$$\begin{array}{*{20}l} y_{i} \sim \mathcal{N}\left(\sigma\delta,\sigma^{2}\right)\\ \delta \sim C(0,r_{i})\\ p\left(\sigma^{2}\right) \propto 1/\sigma^{2} \end{array} $$

where *i* indexes the hypothesis. The null and alternative are then given as

$$\begin{array}{*{20}l} &H_{0}^{\text{OH}}:\delta \sim C(0,r_{0})\\ &H_{1}^{\text{OH}}:\delta \sim C(0,r_{1}) \end{array} $$

To make use of the model, one must specify *r*_0_ and *r*_1_ under both *H*_0_ and *H*_1_, and for *r*_0_→0 and *r*_1_→1 the model recaptures the JZS-prior as a special case. As both hypotheses for *r*_*i*_>0 for *i*=0,1 share some support, the Bayes factor for this model is called the overlapping hypotheses (OH) Bayes factor. Morey and Rouder [41] recommend to use *r*_0_=*r*_1_/10 to establish a narrow equivalence region around the nil value *δ*=0. For details on how to obtain the Bayes factor in this model see Appendix A.

### The non-overlapping hypotheses model

Although the OH model is computationally appealing, it suffers from some problems: First, while in the JZS model there was a clear correspondence between the true effect size and both hypotheses, for the OH model this connection is lost. A true effect of size zero can occur both under the null and alternative hypothesis in the OH model, which troubles interpretation. Even if the true effect size would be known, it would not be possible to decide between *H*_0_ and *H*_1_ with certainty, because both hypotheses share some support, and both share the support of *δ*=0. Consequently, the OH Bayes factor $BF_{01}^{\text {OH}}$ proposed by Morey and Rouder [41] converges for increasing sample size to a nonzero value, even when data were generated under the alternative *H*_1_. To mitigate this problem, Morey and Rouder [41] proposed a second model, the non-overlapping (NOH) hypotheses model:

$$\begin{array}{*{20}l} y_{i} \sim \mathcal{N}\left(\sigma\delta,\sigma^{2}\right)\\ \delta \sim t_{\nu_{0}}\\ p\left(\sigma^{2}\right) \propto 1/\sigma^{2} \end{array} $$

Instead of a Cauchy prior on *δ*, the NOH model assigns the effect size a $t_{\nu _{0}}$ prior with *ν*_0_ degrees of freedom. *ν*_0_=1 yields the JZS Cauchy prior, because the *t*_1_ distribution is equal to the *C*(0,1) distribution. *ν*_0_=*∞* yields a standard normal prior because of the convergence of the $t_{\nu _{0}}$-distribution to a standard normal distribution for *ν*_0_→*∞*. The recommended default value for *ν*_0_ is *ν*_0_=1, because the Cauchy distribution allows for a realistic range of effect sizes for biomedical research [41, 59]^3^. The hypotheses for the NOH model are defined as:

$$\begin{array}{*{20}l} H_{0}^{\text{NOH}}:\delta \sim t_{\nu_{0}} \text{ for }\delta \in (-c,c)\\ H_{1}^{\text{NOH}}:\delta \sim t_{\nu_{0}} \text{ for }\delta \notin (-c,c) \end{array} $$

Morey and Rouder [41] provide an expression for the NOH Bayes factor $BF_{01}^{\text {NOH}}$, which requires only numerical integration to obtain the marginal likelihoods under both $H_{0}^{\text {NOH}}$ and $H_{1}^{\text {NOH}}$. To compute the NOH Bayes factor, the boundaries of the equivalence region have to be determined, that is, the parameter *c*. Morey et al. [41] follow Cohen [34] and use half of a small effect size as the boundaries of the equivalence region, which is equal to (−*c*,*c*)=(−0.1,0.1). Importantly, in the NOH model, both hypotheses are distinct concerning their support, in contrast to the OH model. Additionally, the NOH Bayes factor $BF_{01}^{\text {NOH}}$ converges for increasing sample size *n* to zero under the null hypothesis and to *∞* under the alternative unless the true effect size *δ* is on the boundary *c* or −*c*.

Morey and Rouder [41] even proposed a third model, the so-called hybrid model. Details are provided in Appendix A, and it is not considered in the simulation study later as the model has several drawbacks compared to the OH or NOH models.

### Informed Bayes factors for equivalence testing

A second class of approaches goes back to Gronau et al. [31] and Van Ravenzwaaij et al. [44]. Gronau et al. [31] proposed a parameterization based on the grand mean *μ* and the standardized effect size *δ*, in which case the two-sample Bayesian t-test is modelled as $Y_{ij} \sim \mathcal {N}(\mu _{j},\sigma ^{2})$ for *i*=1,...,*n*_*j*_,*j*=1,2, where *μ*_*j*_=*μ*+(−1)^*j*+1^*σδ*/2. Gronau et al. ([31], Theorem A.1) derived the two-sample likelihood based on the grand mean and the effect size as well as the marginal likelihood *p*(*d*|*H*_0_) under *H*_0_, where *H*_0_:*δ*=0 (see Corollary A.1.2 in the supplementary material of Gronau et al. [31]) and showed that the Bayes factor *BF*_10_ of *H*_1_:*δ*≠0 against *H*_0_:*δ*=0 is given as

2 $$\begin{array}{*{20}l} BF_{10}(t)=\frac{\int T_{\nu}(t|\sqrt{n}_{\delta} \delta)\pi(\delta)d\delta}{T_{\nu}(t)} \end{array} $$

Here, *T*_*ν*_(*t*|*a*) denotes the density of a *t*-distribution with *ν* degrees of freedom and noncentrality parameter *a*. To obtain this Bayes factor, Gronau et al. [31] used the prior *π*_0_(*μ*,*σ*)∝1/*σ*. Consequently, researchers can obtain a Bayes factor based on any proper prior for the standardized effect size *δ* by inserting the prior density of interest for *π*(*δ*). Gronau et al. [31] proposed to use a *t*-prior, and other options include a Cauchy or normal prior [28].

However, the model of Gronau et al. [31] is concerned with the nil hypothesis *H*_0_:*δ*=0, and Van Ravenzwaaij et al. [44] argued that the Bayes factor *BF*_10_ of Gronau et al. which is based on the idea of shifting the centre *μ*_*δ*_ of the Cauchy prior *C*(*μ*_*δ*_,*γ*_*δ*_) away from zero while allowing for varying scale *γ*_*δ*_ could also be used for equivalence testing^4^. Van Raavenzwaaij et al. [44] reasoned as follows: “it is possible to calculate a Bayes factor for the same band around *δ*=0 of 2*c*, but there is no need as the evidence in favor of *δ*=0 can be quantified directly. Because of this, the Bayes factor approach simplifies testing for equivalence, such that no arbitrary band needs to be established.”Van Ravenzwaaij ([44], p. 6)

Consequentially, they reject using a an interval hypothesis at all, as the Bayes factor *BF*_01_ can express evidence for the nil hypothesis already. What is more, the argument of Van Ravenzwaaij against using an equivalence region is that an interval estimate may be *entirely* located inside such a region, but may simultaneously *exclude* the nil value. As a consequence, Van Ravenzwaaij [44] reasoned that it is more useful to employ nil hypothesis testing directly. However, we reject this argument because of the two reasons given below.

First, values inside the equivalence region are interpreted as *practically equivalent* (see also the region of practical equivalence approaches detailed below). As a consequence, one cannot separate between values inside the equivalence region (for practical purpose), and the values *δ*=0.01 and *δ*=0.09 are interpreted as equivalent to *δ*=0 when the equivalence region around *δ*=0 is defined as |*δ*|≤0.1. Therefore, the paradox of an interval estimate being located entirely inside the equivalence region but excluding the nil value only occurs if it is indeed possible to separate between values inside the equivalence region and the nil value. In these cases, anyhow, it would be mandatory to choose a narrower equivalence region, because if it is possible to separate between values inside the equivalence region it is too large for the context of research. If the equivalence region is narrowed until all parameter values inside are interpreted as equivalent for practical purposes, the problem disappears because *all* values inside the equivalence region are interpreted as practically equivalent to the nil value.

Second, we do not agree with Van Ravenzwaaij et al. [44], because the evidence for a nil hypothesis *H*_0_:*δ*=0 is not the same as evidence for a null hypothesis *H*_0_:*δ*∈(−*c*,*c*) for a fixed boundary *c*. Evidence, here, is totally abstract although in practice it would be quantified as a necessary change in belief towards one of both hypotheses via the Bayes factor for example, or the posterior probability. In general, it can be assumed that the evidence obtained differs for the nil and interval hypothesis even when the same data is used. This claim is backed up by the results of the simulation study discussed later in this paper.

Van Ravenzwaaij et al. [44] also provide an interval Bayes factor based on the idea that the Bayes factor as given in Eq. (2) can be extended to interval hypotheses. Details can be found in Appendix A.

In summary, the derivations show that the model proposed by Van Ravenzwaaij is identical to the NOH model proposed by Morey et al. [41] when interval hypotheses are considered, and influenced by the original solution of Gronau et al. [31] for the nil hypothesis *δ*=0. As a consequence, solely the NOH solution of Morey and Rouder [41] is reported for testing based on interval Bayes factors, but notice that the solution obtained via the approach of Van Ravenzwaaij et al. [44] is identical. Also, the nil hypothesis test result via the Bayes factor *BF*_01_ for *H*_0_:*δ*=0 is reported to analyze if the reasoning of Van Ravenzwaaij et al. [44] is legitimate^5^. In this case, the JZS Bayes factor of Rouder et al. [28] is recaptured for *μ*_*δ*_=0 (see Gronau et al. [31]), and this setting is used in the simulation study to simplify comparisons and because *μ*_*δ*_=0 is a reasonable nil value.

### The region of practical equivalence (ROPE)

The approaches to Bayesian equivalence testing presented so far were all based on the Bayes factor. The second branch of proposals does not employ the Bayes factor but focusses on measuring the location of a Bayesian interval estimate like credible or highest-posterior-density (HPD) interval inside the region of practical equivalence, the ROPE. The concept of an interval hypothesis (ROPE) was independently proposed in a wide range of scientific domains, compare Westlake [66], Kirkwood and Westlake [67], Carlin and Louis [68], Hobbs and Carlin [69], Schuirmann [70], Kruschke [58], Lakens [37] and Kelter [32]. Conceptually, it equals the interval hypothesis in the models of Morey et al. [41] and Van Ravenzwaaij et al. [44].

### The ROPE

As detailed above, the region of practical equivalence was proposed independently in a variety of scientific domains under different names *“such as indifference zone, range of equivalence, equivalence margin, margin of noninferiority, smallest effect size of interest, and good-enough belt”* as Kruschke ([36], p. 272) notes. In the two-sample setting, the ROPE was championed, in particular, by Kruschke [58] and Kelter [32, 71]. Starting from the posterior distribution of the parameter of interest, researchers should interpret values inside the region of practical equivalence (ROPE) as equivalent for practical purposes to the value the ROPE is defined around. For example, when conducting a clinical trial which compares the heartbeats per minute of patients in two groups, one could define that the difference of means *μ*_2_−*μ*_1_ is practically equivalent to zero if it lies inside the ROPE [−3,3]. That means a difference of three or fewer heartbeats per minute is interpreted as *practically equivalent to zero*. If the posterior distribution of *μ*_2_−*μ*_1_ now is entirely located inside the ROPE [−3,3], the difference *μ*_2_−*μ*_1_ is interpreted as practically equivalent to zero a posteriori. On the other hand, if the total probability mass of the posterior distribution *μ*_2_−*μ*_1_ is located outside the ROPE [−3,3], the null hypothesis *μ*_2_=*μ*_1_ of no difference can be rejected. The same procedure can be applied to any parameter, *θ* of interest, where for the two-sample t-test, *θ*=*δ*, the effect size. If the probability mass of the posterior lies partially inside and outside the ROPE, the situation is inconclusive.

### The 95% and 100% ROPE

There are two versions of the ROPE, one in which the 95% Highest-Posterior-Density-Interval (HPD) is used for the analysis (95% ROPE), and one in which the full posterior distribution is used (full ROPE). For the effect size *δ*, Kruschke [72] proposed to use [−0.1,0.1] as the ROPE for the null hypothesis *H*_0_:*δ*=0 of no effect, which is half of the effect size necessary for at least a small effect according to Cohen [34] (a small effect is defined as 0.2≤*δ*<0.5 or −0.5<*δ*≤−0.2 according to Cohen [34]). This is essentially the same proposal which was made by Morey et al. [41] independently.

### The support interval ROPE

Both the 95% and the 100% ROPE are based on Bayesian HPD intervals. However, HPD intervals suffer from the problem that they may include values which have not been corroborated by observing the data. Stating such values in a Bayesian interval estimate like an HPD is questionable, which is why Wagenmakers et al. [73] proposed the support interval recently. In this paper, the ROPE approach is extended from standard HPD intervals to the support interval as follows: The support interval ROPE is based on the *BF*=*k* support interval, which consists of all parameter values *θ*, which fulfill *p*(*θ*|*x*)/*p*(*θ*)>*k*. This can be interpreted that values inside the *BF*=*k* support interval have been corroborated by the data by at least a factor *k*. As a default value for *k* for the support interval, Wagenmakers et al. [73] proposed *k*=1 because then the resulting *BF*=1 interval contains precisely those parameter values *θ* which yield a Bayes factor *BF*_01_ larger than one. The equivalence test based on the ROPE and *BF*=1 interval proceeds identically to the situation in which a 95% or 100% HPD interval is employed: If the *BF*=1 support interval is located entirely inside the ROPE, the null hypothesis described via the ROPE is confirmed. If the *BF*=1 support interval is located entirely outside the ROPE, the null hypothesis *H*_0_ described via the ROPE is rejected^6^. In cases where the support interval crosses the ROPE boundaries, the situation remains inconclusive and more data is required. The 95% version of the *BF*=1 support interval is omitted because all values which have been corroborated by observing the data should be located in the ROPE to confirm the null hypothesis.

## Methods

The previous sections showed that the principal approaches to Bayesian equivalence testing consist of solutions based on the Bayes factor and the ROPE. However, no matter which approach is chosen, researchers need to choose the equivalence region (that is, the parameter *c* which determines the interval hypothesis width or the boundary of the ROPE) and hyper-parameters in the prior distributions^7^. The selection of equivalence region boundaries is a major challenge to (Bayesian) equivalence testing approaches and needs to be justified carefully. The following example illustrates the challenge to determine the equivalence region in practice.

### An illustrative example: exhaled volume for lung cancer patients with different tumour sizes

Zieba et al. [74] investigated the post-operative life expectancy of lung cancer patients. Data was collected at Wroclaw Thoracic Surgery Centre for patients who underwent major lung resections for primary lung cancer in the years 2007 to 2011. The sample consists of *n*=470 patients and includes various attributes, among others the size of the original tumour from OC11 (smallest) to OC14 (largest) and the volume that has been exhaled at the end of the first second of forced expiration. While the original study investigated the post-operative life expectancy, here the data is used to study the difference in exhaled volume between patients with different tumour sizes. Clearly, assuming that a precise nil difference exists is unrealistic, so an equivalence test is more appropriate. Even if a two-sample Welch’s t-test is conducted to compare the means of exhaled volume between patients with OC11 and OC12 tumour size classification (with respective group sizes *n*=177 and *n*=257), the result turns out to be non-significant with *t*=−1.0731, 368.94 estimated degrees of freedom and a *p*-value of *p*=0.2839. However, absence of evidence is no evidence of absence so it is not possible to conclude that no difference exists. In order to conduct a (Bayesian) equivalence test, the equivalence region needs to be determined first. Ideally, one would like to use a formal power analysis or use subject-domain knowledge or results from prior studies to set reasonable bounds for the equivalence region. Still, often none of these options is available because subject-domain knowledge does not suggest specific boundaries and no prior research results exist. Then, it remains unclear how to select the equivalence region in an objective manner without resorting to weakly justified default values like *c*=0.10 for the effect size *δ*.

### Boundary selection of equivalence regions in (Bayesian) equivalence tests

In this section, a new proposal is made how to determine the equivalence region for Bayesian equivalence tests in practice. Note that the proposal deals primarily with equivalence tests. However, the interval Bayes factor and the ROPE can easily be used for Bayesian superiority tests, too, although these are not studied in this paper. For example, a ROPE can be selected as [*c*,*∞*) for some $c\in \mathbb {R}$ to resemble a superiority test of *H*_0_:*θ*≥*c* against its alternative, and the interval Bayes factor could be extended to use an interval hypothesis (*c*,*∞*) in the same way. Inferiority tests would work accordingly. However, the simulation study deals only with Bayesian equivalence tests^8^. The results will be used later to implement the new proposal made in this section, and reanalyse the illustrative example.

Regarding the choice of the equivalence region, Morey and Rouder [41] stressed: “Choices of the equivalence regions and weights of the point nil reflect reasoned beliefs about the problem at hand. In fields where interesting effects are smaller (...) the width of the null region may be (...) small. In other fields, where interesting effect sizes are larger (...) the region may be made larger to suit. The task of selecting boundaries is simplified somewhat by the parameterizations. The models are parameterized with respect to standardized effect size. General guidelines already exist (Cohen, 1988), and we note that many journals require reporting some measure of effect size.”Morey and Rouder ([41], p. 25-26)

For a variety of quantities used in biomedical research widely accepted standards exist how to interpret different magnitudes of these quantities. Examples are effect sizes, which have a tradition of being categorized in the biomedical, social and psychological sciences, see Cohen [34]. For effect sizes, a widely accepted ROPE *R* around a null hypothesis *H*_0_:*δ*=0 is given as *R*=[−0.1,0.1], whose boundaries *δ*=−0.1 and *δ*=0.1 are half of the magnitude necessary for at least a small effect as defined by to Cohen [34]. Both Kruschke [58] and Morey and Rouder [41] proposed this default ROPE on *δ*^9^. However, the range of proposals how to select the equivalence region (no matter if for frequentist or Bayesian equivalence tests) is broad, and below only the most established options with a focus on the biomedical sciences are outlined briefly:

(i) According to Lakens et al. [38], researchers often know better which sample sizes are attainable in their field of work than which effect sizes can expected to be observed in a study. As the amount of data available limits the effect size which can be detected, researchers can derive the smallest effect size which they can detect after selecting a test level *α* and their sample size *n* and use this smallest detectable effect size as the equivalence boundary. Note that although it seems that this method primarily applies to frequentist tests because the Bayesian paradigm contains no concept of a type I error, the results of the simulation study presented below will allow to use this method also for Bayesian equivalence tests.
(ii) The U.S. Food and Drug Administration has recommended equivalence bounds for establishing bioequivalence [75], for a discussion see Senn [76].
(iii) Cook et al. [77, 78] proposed three methods: The anchor method for determining the minimally clinical important difference (MCID), where the judgement of relevant stakeholders is used, compare Jaeschke et al. [79]. The distribution method, where both the standard error of a measurement and the smallest detectable difference of a statistical test is employed. The health economic method which asks which effect is necessary in “health units” to justify the amount of money spent for the treatment or therapy.
(iv) Weber and Popova [80] recommended to incorporate meta-analyses to determine the equivalence region.
(v) Simonsohn [81] proposed to set the equivalence boundary at the effect size which a previous study would have had ≈33*%* power to detect. For details see also Lakens et al. [38].
(vi) Ferguson [82], Beribisky, Davidson and Cribbie [83] and Rusticus and Eva [84] argued for incorporating pilot studies to determine the equivalence region.
(vii) Other approaches and examples which select the equivalence region based on prior research are given in Perugini, Gallucci & Constantini [85] and Kordsmeyer and Penke [86].
(viii) In case none of the other justifications of equivalence boundaries is possible, Maxwell, Lau and Howard [87] proposed to use a trivially small value like an effect size of *δ*=0.10 according to Cohen [34]^10^.
(ix) Kruschke [36] provides an in-depth discussion of selecting the boundaries for the ROPE in the Bayesian approach.
(x) Finally, *“the ideal specific meaningful effect should be made through a multi-faceted decision-making process”* ([83], p. 5), see also Rogers et al. [88].

Now, in addition to these proposals another one is made: To use objective criteria like the type I error rate, power and robustness to the prior selection to determine the equivalence region (or to decide between available Bayesian equivalence tests). This has the advantage that it is a stronger justification than using recommended default values such as *δ*=0.1 – see point (viii) – and it can easily be combined with the other approaches. For example, method (i) can be used to select a desired type I level *α* and specify the attainable sample size *n* in the frequentist paradigm. The results of the simulation study presented in this paper allow to use this method for Bayesian equivalence tests, too. They enable to determine which equivalence region is compatible with these desiderata and which power is attained. While it may be the case that the equivalence region compatible with the desired objective criteria is too broad or narrow, this approach allows to judge the consequences of selecting an equivalence region more objectively. Also, if prior research or pilot studies strongly recommend a specific equivalence region – see approaches (iii)-(vi) – the results can be used to investigate the resulting type I error rate and power when selecting this equivalence region and pick the Bayesian equivalence test with the best properties for a specified equivalence region and prior distribution.

### Design of the simulation study

To use the new method for equivalence region selection, a simulation study was performed to analyze the behaviour of the different approaches to Bayesian equivalence testing in the setting of Welch’s two-sample t-test. This section details the design of the simulation study. The next section presents the results and the section thereafter discusses these and shows how to apply them in practice by revisiting the illustrative example.

Pairs of data were simulated which consist of two samples, one for each group, both of which are normally distributed. Four settings were selected to investigate the sensitivity of the approaches: In the first setting, no effect was present, and both groups were identically distributed as standard normal $\mathcal {N}(0,1)$. This allows studying the type I error rate produced by each of the approaches presented in the previous sections. In the second setting, a small effect was present, and the first group was simulated as $\mathcal {N}(2.89,1.84)$ and the second group as $\mathcal {N}(3.5,1.56)$, resulting in a true effect size of

3 $$\begin{array}{*{20}l} \delta=\frac{(2.89-3.5)}{\sqrt{\left(\left(1.84^{2}+1.56^{2}\right)/2\right)}}\approx -0.357 \end{array} $$

In the third simulation setting, a medium effect was present. The first group was generated according to a $\mathcal {N}(254.08,2.36)$ distribution, and observations in the second group followed a $\mathcal {N}(255.84,3.04)$ distribution, resulting in a true effect size of

4 $$\begin{array}{*{20}l} \delta=\frac{(254.08-255.84)}{\sqrt{\left(\left(2.36^{2}+3.04^{2}\right)/2\right)}}\approx -0.646 \end{array} $$

The last setting modelled data in the first group as $\mathcal {N}(15.01,3.4)$ and in the second group as $\mathcal {N}(19.91,5.8)$, which yields a true effect size of

5 $$\begin{array}{*{20}l} \delta=\frac{(15.01-19.91)}{\sqrt{\left(\left(3.4^{2}+5.8^{2}\right)/2\right)}}\approx -1.03 \end{array} $$

For each of the four effect size settings, 1000 datasets following the corresponding group distributions as detailed above were simulated. This procedure was repeated for different samples sizes *n*, ranging from *n*=10 to *n*=200 in steps of size 10 to investigate the influence of sample size *n* on the different approaches. For the equivalence testing approaches based on Bayes factors, the Bayes factor *BF*_01_ was computed for each data set. The equivalence testing approaches based on the ROPE were also computed for each data set. First, for each data set the overlapping hypotheses Bayes factor $BF_{01}^{\text {OH}}$ was computed via transitivity by employing two JZS Bayes factors as detailed in Appendix A. The Cauchy prior width *r*_0_ under the null hypothesis was selected as a tenth of the Cauchy prior width *r*_1_ under the alternative in all simulations. Three settings $C(0,1/\sqrt {2}), C(0,1)$ and $C(0,\sqrt {2})$ were chosen under $H_{1}^{\text {OH}}$ which are based on the recommendations of Rouder et al. [28] and Kelter [40]. The corresponding priors under the null hypothesis $H_{0}^{\text {OH}}$ in the OH model are then given as $C(0,1/(\sqrt {2}\cdot 10)), C(0,1/10)$ and $C(0,\sqrt {2}/10)$.

Second, the non-overlapping hypotheses Bayes factor $BF_{01}^{\text {NOH}}$ was computed according to the numerical integration routine given in Morey et al. [41]. The hyper-parameter *ν* was chosen as *ν*_0_=1 and the scale of the resulting Cauchy prior on *δ* was selected as $1/\sqrt {2}$, 1 and $\sqrt {2}$ to make the results of the OH model and NOH model comparable (for details on the relationship between the $t_{\nu _{0}}$-prior and the Cauchy prior *C*(0,*γ*) on *δ* see the Appendix A in Morey et al. [41]).

Notice that the informed Bayes factor for equivalence testing proposed by Van Ravenzwaaij et al. [44] using the default hyper-parameters *μ*_*δ*_=0 with varying Cauchy scales $\gamma =1/\sqrt {2}, \gamma =1$ and $\gamma =\sqrt {2}$ was not computed for each data set, because it yields identical results as the NOH model of Morey et al. [41] (interested readers can check this in the provided replication script provided at the Open Science Foundation under https://osf.io/2cs75/).

Fourth, the 95% and 100% ROPE equivalence tests based on the standard HPD interval were computed for each data set, and subsequently, the ROPE equivalence test based on the (100%) *BF*=1 support interval was conducted.

All simulations were repeated for three different ROPEs: The recommended default ROPE [−0.1,0.1] around *δ*=0, a narrower ROPE of [−0.05,0.05] and a slightly wider ROPE [−0.15,0.15]. This allows judging the influence of the ROPE itself on the obtained results next to the influence of the prior elicitation and sample size. The ROPEs were selected to include the widely recommended default choice *R*=[−0.1,0.1], as well as a larger and smaller one. ROPEs of substantial size (e.g. [−0.4,0.4]) are of less interest, as the use of accepting a very wide interval hypothesis (like *H*_0_:*δ*∈[−0.4,0.4]) is of limited use in practice. Also, effects like *δ*≥0.2 would already be categorized as small according to Cohen [34], so a ROPE of [−0.2,0.2] would already include effects which are often already regarded as non-negligible.

The quantities of interest in the simulations were the type I and type II errors, the power and robustness to the prior modeling. Also, the total error rate was of interest. While formally Bayesian statistical theory has no concept of type I or II error, a Bayes factor *BF*_01_<3 (or *BF*_10_≥3) was interpreted as a false-positive result when the true effect size *δ* was zero. Similarly, if an effect was present (no matter if small, medium or large), a Bayes factor of *BF*_01_≥3 (or *BF*_10_<3) was interpreted as a false-negative result, a type II error. The threshold reflects at least moderate evidence for or against a hypothesis according to conventional Bayes factor scales [33, 60].

A result based on the 95% ROPE or 100% ROPE equivalence test using an HPD or support interval was interpreted false-positive when it was located completely outside the corresponding ROPE around *δ*=0, although the true effect size is zero. Similarly, if the HPD or support interval was located entirely inside the ROPE but the effect size was nonzero, this was interpreted as a type II error.

The percentage of type I and II errors was computed as the number of significant results divided by *n*=1000. This is a Monte Carlo estimate for the type I and II error probabilities of the different Bayesian equivalence testing approaches and a quantity crucial for making research reproducible [89]. The sum was calculated as a Monte-Carlo estimate for the total error rate of a method.

As solutions based on the ROPE only require a posterior distribution *p*(*δ*|*x*) of the effect size, for all results the corresponding posterior *p*(*δ*|*x*) of the NOH model of Morey and Rouder [41] was used based on 5000 MCMC draws, which is implemented in the BayesFactor R package [90]. This ensures that differences in the obtained results are not caused by the different statistical models on which the posterior distribution is based^11^. The ROPE indices were computed via the bayestestR package [47], and the OH and NOH Bayes factors of Morey et al. [41] were computed via the BayesFactor R package [90]^12^.

The statistical programming language R [91] was used for the simulations. A commented replication script which reproduces all results and figures is provided at the Open Science Foundation at https://osf.io/2cs75/.

## Results

This section provides the results of the simulation study. Four subsections provide answers to the four research questions formulated above. First, the influence of sample size on type I errors is analysed.

### Type I error rates and influence of sample size

**Fig. 1:**
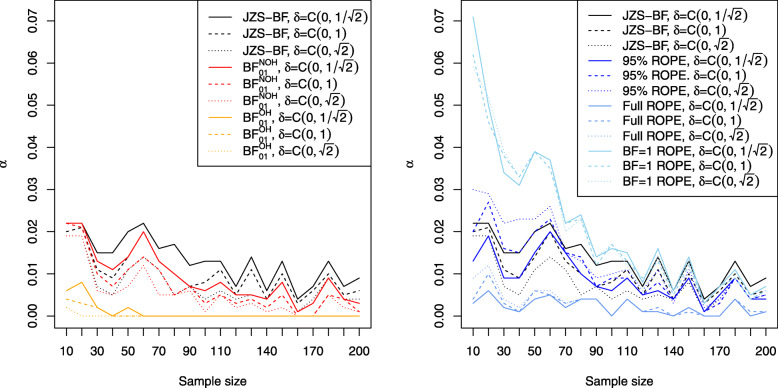
Influence of sample size *n* on the type I error rate attained by Bayesian equivalence approaches based on the Bayes factor (left) and the ROPE (right); the default equivalence region *R*=[−0.1,0.1] is used in all settings

This section analyses the first part of the first research question: Which type I error rates are attained by the various available Bayesian approaches to equivalence testing and how do the obtained type I error rates depend on sample size? Figure 1 shows the resulting type I error rates for Bayesian equivalence testing approaches which are based on the Bayes factor (left plot) and the ROPE (right plot). Depending on the sample size *n*, the error rates differ. The solid lines correspond to a medium $C(0,1/\sqrt {2})$ prior, the dashed lines to a wide *C*(0,1) prior, and the dotted lines to an ultrawide $C(0,\sqrt {2})$ prior. The black lines correspond to the JZS Bayes factor of Rouder et al. [28], which tests the precise null hypothesis *H*_0_:*δ*=0 against *H*_1_:*δ*≠0 to compare the equivalence testing approaches with this approach, too. For increasing sample size *n*, the Bayes factors *BF*_01_ converge to *∞* because of the consistency of the Bayes factor^13^. As a consequence, the type I error rates *α* converge to zero, too. However, the speed of this convergence can be slow, and the left plot in Fig. 1 reveals that the solutions based on the Bayes factor achieve type I error rates of about 0.02 or less. For about *n*=200 samples in each group, the type I error rate is reduced to approximately *α*=0.01 or less. An exception is given by the OH model of Morey et al. [41], which attains a type I error rate of zero for about *n*≥60, no matter which prior is used on *δ*.

The right plot in Fig. 1 shows the situation for the approaches based on the ROPE: First, for small sample sizes the approaches based on the ROPE yield larger type I error rates. Second, the *BF*=1 support interval ROPE yields the largest type I error rates of all ROPE approaches. The 95% ROPE yields also larger type I error rates than the full ROPE, which achieves the best type I error control. While the approaches based on the ROPE achieve an inferior type I error control for small sample sizes compared to the approaches based on the Bayes factor, for increasing sample size *n*, that is, for about *n*≥120 samples in each group the error rates are similar to the ones of the approaches based on the Bayes factor. Additionally, the full ROPE is an exception: It controls the type I error rate even for small sample sizes like *n*=10 or *n*=20 below *α*=0.01, making it an attractive option among the ROPE-based approaches to Bayesian equivalence testing.

### Power analysis and type II error rates

This section provides answers to the second research question: Which sample size is necessary for a selected Bayesian equivalence testing approach to detect a prespecified (e.g. small, medium or large) effect size?

**Fig. 2:**
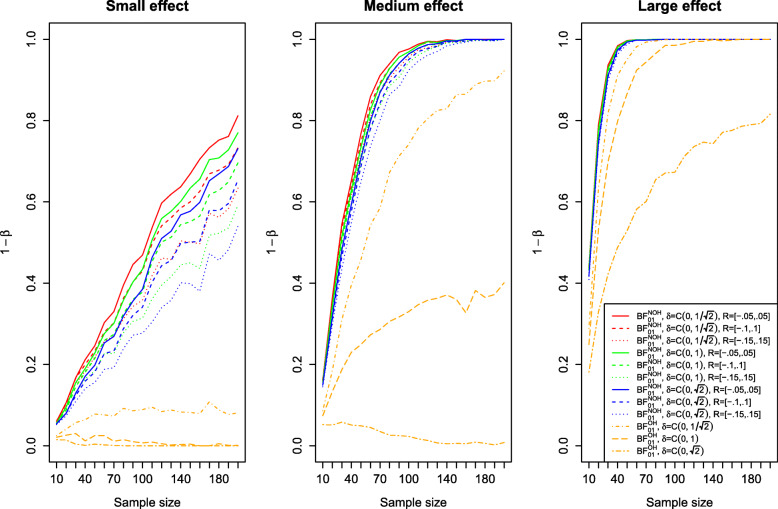
Power analysis for the Bayesian equivalence testing approaches based on the Bayes factor for small, medium and large effect sizes

Figure 2 shows the results for the Bayesian equivalence testing approaches based on the Bayes factor: The left, middle and right plots show the results for a small, medium and large effect size *δ*, as specified in the details about the simulation study. First, for small effects no approach achieves a power larger than ≈80*%*, that is a small effect is detected only with 80% probability, even when *n*=200 samples are used in each group. However, the differences between the various approaches are profound. For the NOH model the power ranges from ≈60−70*%* when the standard ROPE *R*=[−0.1,0.1] is used and *n*=200 samples are observed in each group (shown as the dashed lines under the three different prior settings), while for the narrower or wider ROPE the attained power varies accordingly. Notice that the power of the OH model of Morey et al. [41] (which had a superior type I error control compared to the other approaches as shown in Fig. 1) lacks sufficient power to detect small effects even for large sample sizes *n*. Even for *n*=200 samples in each group, the OH model achieves a power of less than 10%. Second, for increasing effect size *δ*, the power of all approaches increases, which is to be expected. For medium effect sizes, all approaches based on the Bayes factor except for the OH model of Morey et al. [41] achieve a power of ≈80*%* for *n*=60 samples in both groups. For *n*≥100 samples in each group, the power is close to 90−95*%*. For large effects, even *n*=20 samples suffice in each group to achieve a power of 80% as shown in the right plot of Fig. 2.

**Fig. 3:**
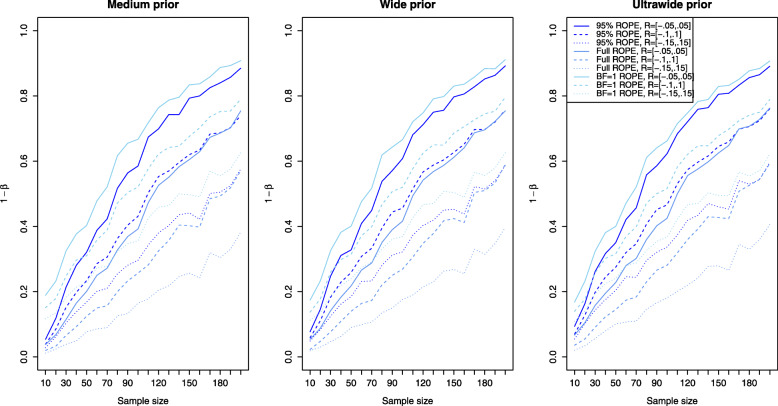
Power analysis for the Bayesian equivalence testing approaches based on the ROPE for an underlying small effect size

**Fig. 4:**
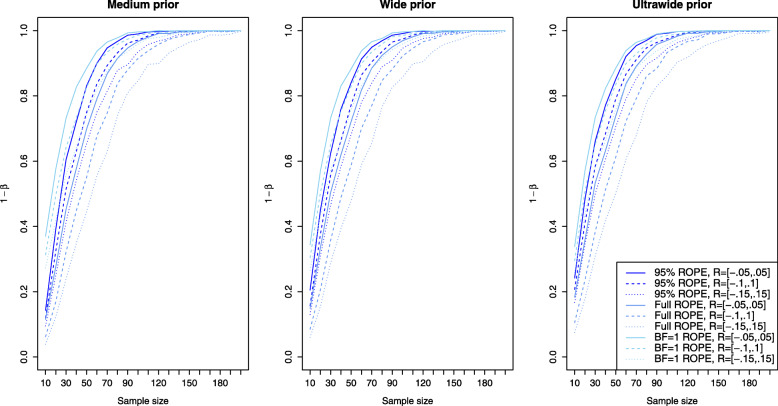
Power analysis for the Bayesian equivalence testing approaches based on the ROPE for an underlying medium effect size

**Fig. 5:**
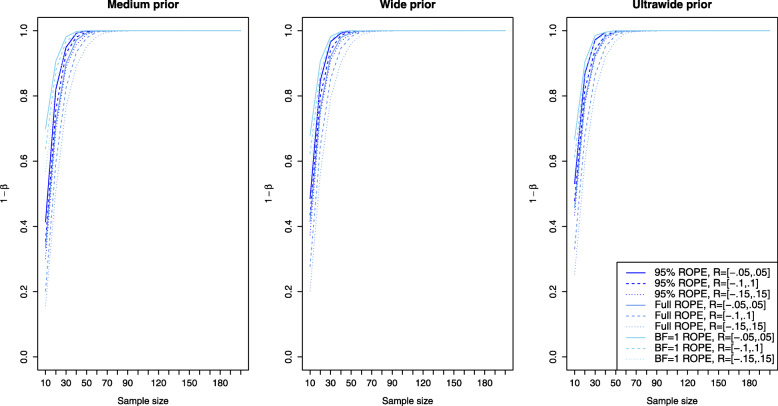
Power analysis for the Bayesian equivalence testing approaches based on the ROPE for an underlying large effect size

Figure 3 shows the results for the simulation setting of a small effect size *δ* for the ROPE-based approaches. Now the left plot corresponds to the results obtained via the medium prior and the middle and right plots correspond to the results obtained by the wide and ultrawide prior on the effect size. In all three plots, the results are shown for small effect size. Compared to the power of the approaches based on the Bayes factors for a small effect (shown in the left plot of Fig. 2), the power of the ROPE-based approaches is similar. For *n*=200 samples in each group, a power of approximately 60−80*%* is attained for the default ROPE *R*=[−0.1,0.1] depending on the prior chosen (see the dashed lines). The ROPE and the prior selected play an important role in attaining power as indicated by Fig. 3. Figures 4 and 5 show the results for the power of the ROPE-based approaches to Bayesian equivalence testing when a medium and large effect is present. From Fig. 4 it is clear that no matter which prior or ROPE is chosen, *n*≥90 samples in each group suffice to reliably detect a medium effect with a power of about 80%. Figure 5 even shows that for a large present effect, *n*≥30 samples suffice to achieve a power of approximately 80%. While Fig. 1 demonstrated that the *BF*=1 support interval ROPE yielded the largest type I error rates, Figs. 3, 4 and 5 show that the *BF*=1 support interval ROPE approach achieves the largest power (or equivalently, smallest type II error rate). The 95% ROPE follows after that, and the approach based on the full ROPE attains the smallest power. The results show, that, in general, the full ROPE is the most cautious approach concerning the type I error control at the price of a smaller power, while the *BF*=1 support interval is positioned at the other end of the spectrum: It has the largest type I error rate but best power of the ROPE-based approaches.

### Influence of prior modeling

This section presents answers to the third research question: How robust are the different Bayesian approaches to equivalence testing concerning the prior modeling? Figures 1, 2, 3, 4 and 5 provide insights to this question, and we start with the Bayes factor based approaches. From Fig. 1 one can observe that for increasing prior width *γ* in the Cauchy prior *C*(0,*γ*) on *δ*, the type I error rate becomes smaller. This is to be expected, because from the Savage-Dickey density ratio [53, 54], the Bayes factor *BF*_01_ can be expressed as the ratio of the ordinate of the posterior density at the nil value *δ*_0_=0 and the prior density at the nil value *δ*_0_=0:

$$\begin{array}{*{20}l} BF_{01}=\frac{p(\delta_{0}|H_{1},x)}{p(\delta_{0}|H_{1})} \end{array} $$

For increasing prior width, the value of the prior density *p*(*δ*_0_|*H*_1_) becomes smaller, and as a consequence, *BF*_01_ becomes larger. As a type I error is defined as a Bayes factor of *BF*_01_<1/3 (or equivalently, *BF*_10_≥3), the type I error rate decreases for increasing prior width *γ*. As a consequence, due to the Jeffreys-Lindley paradox [92], for *γ*→*∞*, the type I error rate converges to zero as the null hypothesis is always accepted.

However, Fig. 2 shows that increasing prior width decreases the resulting power of the Bayes factor-based approaches, too. For example, the left plot in Fig. 2 reveals that for a small effect size, the NOH approach of Morey et al. [41] using an ultrawide $C(0,\sqrt {2})$ prior achieves the smallest power of the three prior settings (see the blue dashed line). Similar observations can be made in the middle and right plots of Fig. 2.

Concerning the approaches based on the ROPE, a reversed phenomenon is observed. From the right plot in Fig. 1 one can see that for increasing prior width the number of type I errors increases. This phenomenon can be explained as follows: When prior width increases, the posterior distribution is less drawn towards zero, allowing the posterior to concentrate farther away from *δ*_0_=0. As a consequence, it becomes easier for the 95% HPD, 100% HPD or *BF*=1 support interval to concentrate entirely outside the ROPE *R*. Therefore, the type I error rate becomes larger when a wider prior is used on *δ*. This phenomenon is visualised in Fig. 1 by the fact that the dotted or dashed lines (which correspond to the ultrawide or wide Cauchy prior setting) are located above the solid lines (which correspond to the medium Cauchy prior setting).

Figures 3, 4 and 5 indicate, that the power of the ROPE based approaches is influenced in the same way: Although it is difficult to spot, the power under an ultrawide $C(0,\sqrt {2})$ prior is always slightly larger than under a wide *C*(0,1) prior, which is again slightly larger than under a medium $C(0,1/\sqrt {2})$ prior.

In summary, in contrast to the Bayes factor based approaches the influence of the prior width on the ROPEs is reversed: Wider priors imply increased power at the cost of more type I errors for the ROPE-based approaches, while for the approaches based on the Bayes factor wider priors imply fewer type I errors at the cost of less power.

### Influence of the interval hypothesis boundaries

Now, the fourth research question aimed at the influence of the equivalence region itself. How does the size of the equivalence region influence the above results? That is, how do type I and II error rates, power and robustness to the prior elicitation vary when the size of the equivalence region is expanded or narrowed?

**Fig. 6:**
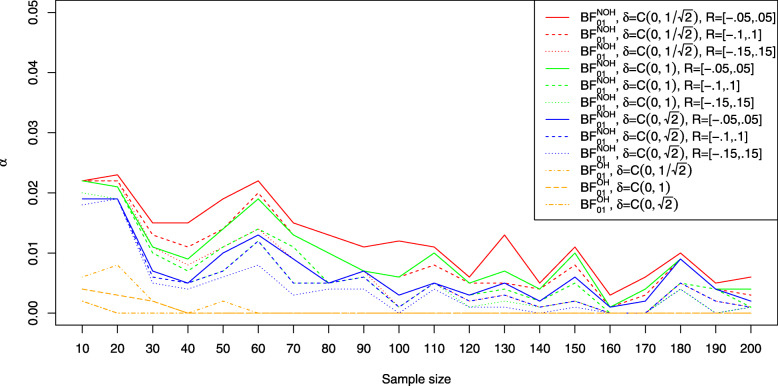
Influence of the equivalence region on the type I error rates for the Bayesian equivalence testing approaches based on the Bayes factor

Figure 6 provides answers for the approaches based on the Bayes factor. The solid lines correspond to the narrower ROPE *R*=[−0.05,0.05], while the dashed lines present the results for the default ROPE *R*=[−0.1,0.1] and the dotted lines show the results for the wider ROPE *R*=[−0.15,0.15]. For wider ROPEs *R*, the associated type I error rate decreases under a fixed prior setting. This can be explained by considering the definition of the Bayes factor itself: The Bayes factor *BF*_01_ is the ratio of the marginal likelihoods *f*(*x*|*H*_0_) and *f*(*x*|*H*_1_). If a wider ROPE *R* is chosen, the marginal likelihood under the null hypothesis *H*_0_:*δ*∈(−*c*,*c*)=:*R* (e.g. in the NOH model of Morey et al. [41]) is increased. As a consequence, *BF*_01_ grows. As a type I error happens whenever *BF*_01_<1/3 (or equivalently, *BF*_01_≥3), the type I error rate becomes smaller for increasing size of the equivalence region (or ROPE) *R* for the Bayes factor based approaches.

Figure 2 presents further insights concerning the power of the approaches which are based on the Bayes factor under varying sizes of the equivalence region. Clearly, for a fixed prior setting the power of the approaches is always smallest under the widest equivalence region *R*=[−0.15,0.15], and largest under the most narrow equivalence region *R*=[−0.05,0.05]. Compare, for example, the solid, dashed and dotted blue lines in Fig. 2. The balance between a reduced type I error rate by increasing the size of the equivalence region and a decreased power is an important aspect when considering the Bayes factor-based approaches to Bayesian equivalence testing.

**Fig. 7:**
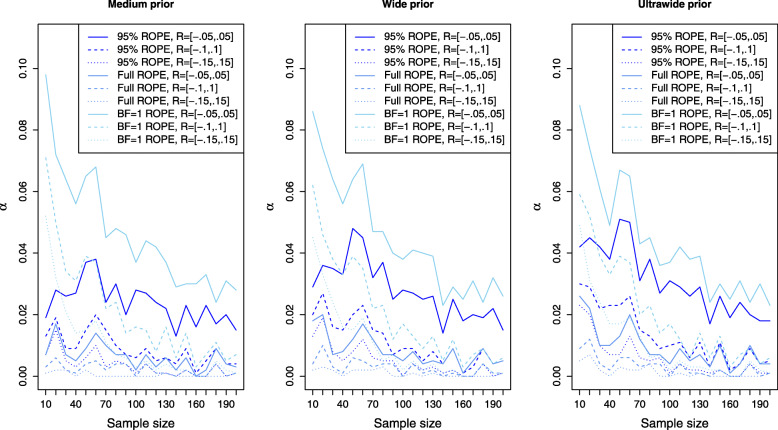
Influence of the equivalence region on the type I error rates for the Bayesian equivalence testing approaches based on the ROPE

Switching to the power of the approaches employing the ROPE, Fig. 7 provides answers. The left plot shows the resulting type I error rates for varying equivalence regions under a medium prior. The middle and right plot shows the type I error rates obtained from different equivalence regions under a wide and ultrawide prior on the effect size *δ*. Clearly, for increasing size of the equivalence region *R*, the type I error rate becomes smaller. This is to be expected, because a larger equivalence region *R* makes it more difficult for the 95% HPD, 100% HPD or *BF*=1 support interval to be located entirely outside the equivalence region *R*. As a consequence, the number of type I errors is smaller for a wider equivalence region.

Concerning the resulting power under varying equivalence region sizes, Figs. 3, 4 and 5 show that larger equivalence regions yield smaller power, and equivalently, more type II errors. For example, the left plot in Fig. 3 which corresponds to a small underlying effect size indicates that the power ranges from ≈40*%* for *R*=[−0.15,0.15] over ≈55*%* for *R*=[−0.1,0.1] to ≈70*%* for *R*=[−0.05,0.05] for the full ROPE and *n*=200 samples in each group, compare the solid, dashed and dotted lines in the left plot of Fig. 3. In general, the relationship is similar to the relationship identified for the Bayes factor based approaches: Both for the Bayes factor based approaches and the approaches based on the ROPE, a reduced type I error rate and decreased power are the consequence of increasing the size of the equivalence region.

## Discussion

The last section presented the results of the simulation study which provided answers to the four research questions formulated in advance. This section discusses the obtained results.

Concerning the first research question, two aspects are important to mention: First, as shown in Fig. 1, the various Bayesian approaches to equivalence testing differ concerning their ability to control the type I error rate. While there are approaches which essentially reduce the number of type I errors to zero even for small sample sizes like the OH model of Morey and Rouder [41], other approaches like the *BF*=1 support interval ROPE achieve even larger type I error rates for small sample sizes than traditional NHST solutions for precise hypotheses (when the significance threshold *α*=0.05 is chosen). As a consequence, it is important to consider the relevance of type I error control for the situation at hand when selecting an approach. A recommended candidate is given by the NOH model of Morey and Rouder [41] under a wide *C*(0,1) prior on *δ* when a Bayes factor-based approach is favoured. The NOH Bayes factor attains good type I error control even for moderate sample sizes. However, if an even better type I error control is desired the full ROPE approach is recommended under the same *C*(0,1) prior. The full ROPE guarantees excellent type I error control in this setting as indicated in the right plot of Fig. 1.

Second, the influence of sample size is important both for the type I error rates and the power of the different approaches. Concerning the type I error rates, for small sample sizes below *n*=20 the approaches based on the ROPE can yield larger type I errors than the Bayes factor-based approaches. This can be relevant, in particular, in biomedical research where often sample sizes are small (e.g. in studies for rare diseases or when recruiting participants is expensive). However, an exception is given by the full ROPE which is recommended in small sample settings.

Notice that all approaches except for the *BF*=1 support interval ROPE and 95% ROPE attain smaller type I error rates than the precise hypothesis test based on the JZS Bayes factor, compare Fig. 1.

Concerning the second research question, the power analysis revealed that there are profound differences between the available approaches, and showed which sample size is necessary for a selected Bayesian equivalence testing approach to detect a prespecified (e.g. small, medium or large) effect. In general, the approaches based on the ROPE and the Bayes factor perform similarly regarding the required sample size to detect an existing effect. However, there are differences between the approaches: The OH model of Morey and Rouder [41] attained a superior type I error control compared to all other Bayes factor-based approaches but lacks sufficient power as shown in Fig. 2. As a consequence, it is not recommended to use this model. Instead, the NOH model is a more balanced alternative, and the results provided in Fig. 2 show which sample sizes are necessary to attain a specific power. Regarding the approaches based on the ROPE, the full ROPE (which offered the best type I error control) yields the smallest power. The *BF*=1 support interval ROPE and the 95% ROPE yield better power. However, this increase in power comes at the price of a higher type I error rate, compare Fig. 1.

Concerning the third research question about the robustness of the different Bayesian approaches to equivalence testing to the prior modeling selected, two points are worth mentioning: First, the prior modeling plays a crucial role to balance the type I error rate and power both for the approaches based on the Bayes factor and the approaches based on the ROPE. For the Bayes factor-based approaches, increasing the prior width reduces the type I error rate but simultaneously decreases the power of the tests. For the ROPE based approaches, the situation is reversed: Increasing the prior width increases the type I error rate but implies a higher power to detect an existing effect.

### Revisiting the illustrative example

Concerning the fourth and fifth research question, the results demonstrated how the size of the equivalence region influences the other results. First, the type I error rate and the power are influenced by the size of the equivalence region both for the approaches based on the Bayes factor and the approaches based on the ROPE. What is more, both for the Bayes factor based approaches and the approaches based on the ROPE increasing the size of the equivalence region yields a reduced type I error rate at the cost of decreased power. This is an important aspect because the consequence is that results based on different equivalence regions are, in general, not comparable. For example, if a result obtained from a wide equivalence region *R*=[−0.15,0.15] shows evidence for the null hypothesis *H*_0_, it can happen that the result based on a narrow equivalence region *R*=[−0.05,0.05] shows evidence for the alternative, because the power is higher in the smaller equivalence region setting. This is no defect of the method but to be expected because changing *R* implies that a different test is carried out. Consequently, this phenomenon underlines how important it is to justify the selected equivalence region.

This leads to the fifth research question and the primary challenge in applying Bayesian equivalence tests in practice: How should the equivalence region be chosen? Based on the results, the new proposal made earlier in this paper can be implemented.

Reconsider the illustrating example of Zieba et al. [74]. The goal was to test for equivalence in exhaled volume between patients with OC11 and OC12 tumour size classification. Although there may be subject-domain knowledge or prior research results available which helps in determining the equivalence region, suppose no such information is available. Suppose further that a type I error rate of 5% is accepted at most, and the desired power is 80% to detect a small effect (up to ≈*δ*=0.35). The narrower the equivalence region can be chosen to fulfill these desiderata the better, as the resulting statement about equivalence then becomes more precise. Suppose further that a wide Cauchy prior *C*(0,1) is chosen which reflects the prior beliefs about the effect size *δ*. Importantly, this prior should not be used as a tuning parameter to attain a specific type I error rate or power, but needs to be selected in advance.

First, consider the Bayes factor solutions: Fig. 6 shows that all models attain a type I error rate of 5%. However, the yellow lines indicate that a larger ROPE like *R*=[−0.15,0.15] will yield smaller type I error rates for *n*=170 (we use the smaller of both group sizes for all comparisons). The left plot in Fig. 2 shows the resulting power of the Bayes factor solutions under the assumption of a small effect. It shows that for *n*=170 samples, the NOH Bayes factor attains a maximum of 70% power under the equivalence region *R*=[−0.05,0.05] (solid yellow line). So, the best solution of the NOH Bayes factors yields *α*≈0.01 and *β*=0.70 for *n*=170 samples under the *C*(0,1) prior and leads to an equivalence region *R*=[−0.05,0.05].

Second, consider the ROPE solutions: The middle plot in Fig. 7 visualizes the resulting type I error rates of the ROPEs under the selected *C*(0,1) prior. For *n*=170 samples, all solutions yield an error rate smaller than *α*=0.05, although the 95% and BF=1 ROPEs yield higher rates than the full ROPE. The middle plot in Fig. 3 shows the resulting power for the ROPEs under the *C*(0,1) prior, and for *n*=170 samples the only options to attain 80% power are the 95% ROPE or BF=1 ROPE with an equivalence region of *R*=[−0.05,0.05] (solid blue lines). The 95% ROPE yields a smaller type I error rate of ≈0.02 as shown in the middle plot of Fig. 7, so to fulfill the objective criteria *α*≤0.05 and *β*≥0.8 the 95% ROPE with an equivalence region of *R*=[−0.05,0.05] is recommended under the *C*(0,1) prior.

In total, the 95% ROPE fulfills both desiderata, while the NOH Bayes factor has only 70% power (but a type I error rate of only ≈0.01 compared to ≈0.02 for the 95% ROPE). Thus, given the objective criteria, the 95% ROPE is the optimal solution with an equivalence region of *R*=[−0.05,0.05].

A Bayesian equivalence test based on the 95% ROPE for *R*=[−0.05,0.05] yields a 95% HPD [−0.05,0.04] for *δ*, which is entirely located inside *R*. Thus, equivalence of exhaled volume between patients with OC11 and OC14 classification is established. The small equivalence region *R*=[−0.05,0.05] shows that the effect is very close to the precise null effect *δ*=0, and the objective criteria guarantee that the type I error rate and power are as desired^14^. The new method thus allows for objective determination of the equivalence region in the illustrating example. The justification is based on statistical criteria like the resulting type I error rate, power, available sample size and robustness to prior selection. Here, the latter should only be used when no prior has already been elicited due to prior research or subject-domain knowledge reflecting the beliefs about the parameter. In particular, it is strongly advised against selecting a prior as a tuning parameter to attain a specific error control: The relevant quantities which can be tuned are the sample size and the equivalence region.

**Fig. 8:**
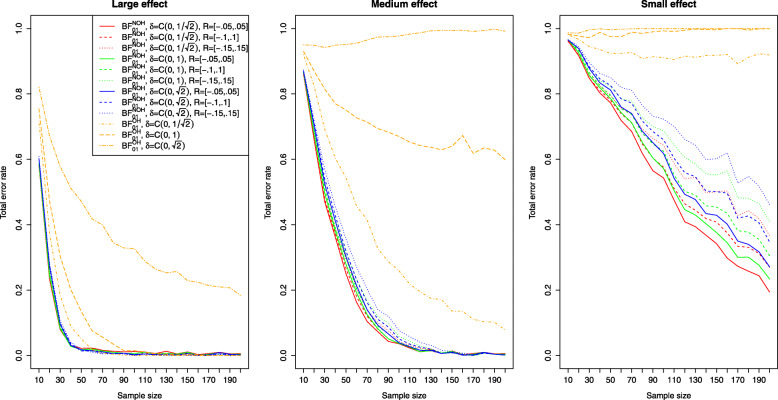
Total error rates for the Bayesian equivalence testing approaches based on the Bayes factor for small, medium and large effect size

**Fig. 9:**
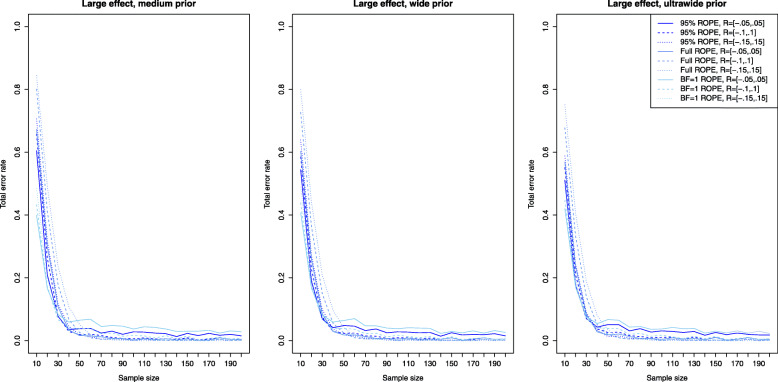
Total error rates for the Bayesian equivalence testing approaches based on the ROPE for an underlying large effect size

**Fig. 10:**
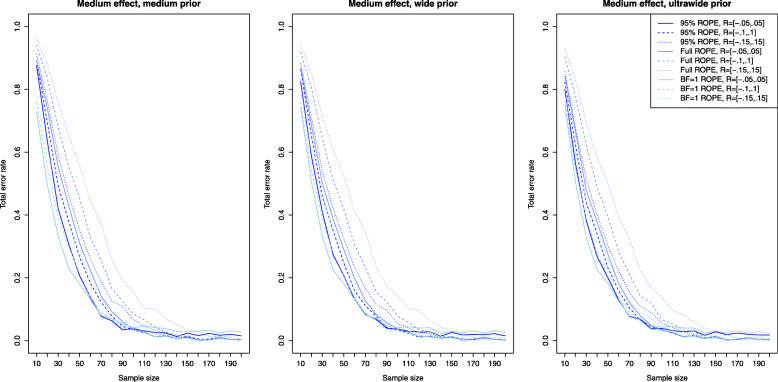
Total error rates for the Bayesian equivalence testing approaches based on the ROPE for an underlying medium effect size

**Fig. 11:**
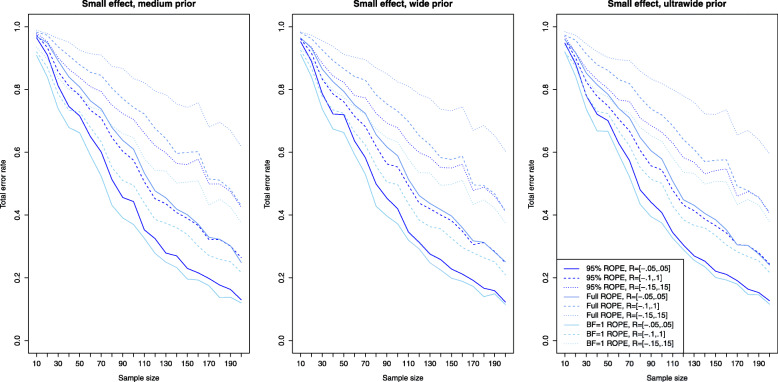
Total error rates for the Bayesian equivalence testing approaches based on the ROPE for an underlying small effect size

As a final note, one could also use the total error rates to select the equivalence region: Figs. 8, 9, 10 and 11 show the total error rates (type I + type II) for the Bayes factor and ROPE solutions. It is important when using this alternative method to acknowledge that the loss incurred by making a type I or II error is assumed to be identical. This often is unrealistic in biomedical research, as the loss incurred through a false-negative or false-positive result often differ. Still, if one supposes that a total rate of misclassification of 20% is acceptable, the right plot in Fig. 8 shows that again, the NOH Bayes factor for *R*=[−0.05,0.05] under the *C*(0,1) prior is the only option, although it has a slightly larger total error rate for *n*=170 (about 30%). The middle plot in Fig. 11 shows the corresponding total error rate of the ROPEs under the *C*(0,1) prior and a small effect. Again, the 95% or BF=1 ROPE for *R*=[−0.05,0.05] are suitable then. Here, using the BF=1 ROPE has the additional advantage of including only parameter values which have been corroborated by observing the study data. The conclusions remain identical and equivalence is established under this alternative approach.

## Conclusion

Null hypothesis significance testing (NHST) remains one of the most widely used methods in the biomedical and cognitive sciences. However, the problems of NHST and *p*-values have been lamented widely and various Bayesian alternatives have been proposed recently. While some of these proposals focus on equivalence testing which aims at testing an imprecise hypothesis instead of a precise point null hypothesis, multiple problems have mitigated more widespread use of Bayesian equivalence tests in practice. This is undesirable because researchers can always expect to observe some (although often negligibly small) effect size because of noise in the data, and the assumption of an interval hypothesis is, as a consequence, more realistic in a variety of biomedical research.

First, the selection of an interval hypothesis, or equivalently, an equivalence region seems arbitrary. Second, several Bayesian approaches to equivalence testing have been proposed which differ both in the underlying theory and computational details.

In this paper, a new proposal was made how to select the equivalence region for Bayesian equivalence tests in the two-sample setting based on objective criteria like the resulting type I error rate, power and robustness to the prior selection. A simulation study investigated how existing Bayesian equivalence tests behave regarding their ability to detect an effect, control type I errors and their robustness to the prior selection, which allows to implement the new proposal in the two-sample setting.

First, the available approaches to Bayesian equivalence testing in the two-sample setting and the selection of the equivalence region for the effect size were discussed. As stressed by Morey and Rouder [41], it is important to establish default equivalence regions for specific parameters and statistical methods in biomedical research: “Certainly if researchers are able to interpret effect size measures in the context of existing literature, it is not difficult to extend this to setting bounds on equivalence regions. Eventually conventions may arise, as they have with type I error rate.”Morey and Rouder ([41], p. 26)

However, it is questionable if there will ever be widely accepted default values for every research context. Instead of focussing on establishing default values, the new proposal how to determine the equivalence region together with the results of the simulation study allow to make Bayesian equivalence testing more objective. Approaches based on such objective criteria were missing by now. In this paper, it was shown that from a mathematical perspective, the default region *R*=[−0.1,0.1] of practical equivalence for the effect size parameter *δ* in the two-sample setting achieves reasonable type I error control and sufficient power to detect a present effect. Simultaneously, the influence of the prior elicitation was only moderate, although not negligible under this default equivalence region, compare Figs. 2, 3, 4, 5, 6 and 7.

Second, results showed that the proposals for Bayesian equivalence testing differ in their sensitivity to the prior modeling, their power, and the associated type I error rates.

Based on the obtained results it was demonstrated via an illustrating example how to choose the equivalence region to attain a specific power and type I error rate. This demonstrated how to implement the proposal in practice.

Additionally, the results showed that the prior selection influences the type I error rate and the power of the tests, both for the approaches based on the ROPE and the approaches based on the Bayes factor. However, it was shown that this relationship is reversed between the two classes of approaches to Bayesian equivalence testing.

Furthermore, it was shown that the size of the equivalence region influences the type I error rates and power of the various available approaches. As a consequence, it is important to justify the selection of an equivalence region, and the method proposed in this paper makes the selection less subjective. By incorporating the results presented, researchers can prevent the claim of subjectivity about a selected equivalence region. In contrast, reporting the corresponding type I error rate and power adds value to a Bayesian equivalence test and justifies the selection of the equivalence region boundaries. Also, it helps to determine the required number of participants if there exist strong a priori reasons to choose a specific equivalence region, for example based on prior research or subject-domain knowledge. Then, the resulting power can be quantified via the approach.

The choice of a method ultimately depends on the criteria which are required to hold. While in each single case, the optimal procedure can be determined via the proposed method and the results provided in this paper, Table 1 provides a general overview of the pros and cons of the competing approaches.

**Table 1:** Comparison of the Bayesian approaches to equivalence testing

	Pro	Con
Interval BFs	+ Influenced more moderately by varying equivalence regions *R*, compare the spread in power between different choices of *R* in Fig. 2 (for example, power ranges between 60% and 80% for small effects)	– Less robust to the prior selection (for example, compare the difference in power depending on the selected prior in Fig. 2)
	+ Recommended in situations with little uncertainty about the prior selection but limited knowledge how to choose the size of the equivalence region	– The OH model may be attractive in some situations but yields very large error rates, making it practically unusable
	+ Reliable type I error control for small sample sizes	
ROPEs	+ Robust to the prior selection (see the horizontal progression in Figs. 3, 4 and 5)	– Influenced stronger by varying equivalence regions *R*, compare the spread in power between different choices of *R* in Figs. 3 and 4 (for example, power ranges from 50% to 80% for small effects and medium prior)
	+ Recommended in situations where the equivalence region is motivated from subject-domain knowledge or pilot studies but there is considerable uncertainty about the prior	– Only the full ROPE controls type I errors for small sample sizes
	+ The full ROPE yields the best type I error control which is important if the stakes of a false-positive result are high	

As Table 1 shows, when prior specification is difficult, the ROPEs resulting error rates and power change less than the ones of the interval Bayes factors. Additionally, the equivalence region can be determined based on the method proposed in this paper using objective criteria. However, if the prior can be elicited relatively straightforward (e.g. there are strong reasons from subject-domain knowledge to assume a specific prior), the interval Bayes factor is less prone to the uncertainty in determining the equivalence regions. Also, interval Bayes factors yield better type I error control in small sample settings, which is important in some settings. On the contrary, the full ROPE yields the best overall type I error control but the smallest power: When stakes of a false-positive are high, the full ROPE is the appropriate solution.

Establishing default equivalence regions for specific domains, like *R*=[−0.1,0.1] on the effect size *δ* in the biomedical and cognitive sciences is often regarded as one of the most important challenges to make Bayesian equivalence testing more attractive for practitioners. However, more research is required to establish default regions of practical equivalence, in particular, for more complex models with a large number of parameters.

In summary, the results provided in this paper can help to make Bayesian equivalence testing more objective by selecting among the existing approaches based on objective criteria like type I error control, the power to detect a given effect size, and robustness to the prior elicitation. Following the recommendations provided in this paper could improve the quality and reproducibility of biomedical research when it comes to Bayesian equivalence testing in the two-sample setting. Importantly, it allows researchers to determine the equivalence region and choose among the available Bayesian equivalence tests based on objective criteria.

## Appendix A

### Overview about frequentist approaches to equivalence testing

**Fig. 12:**
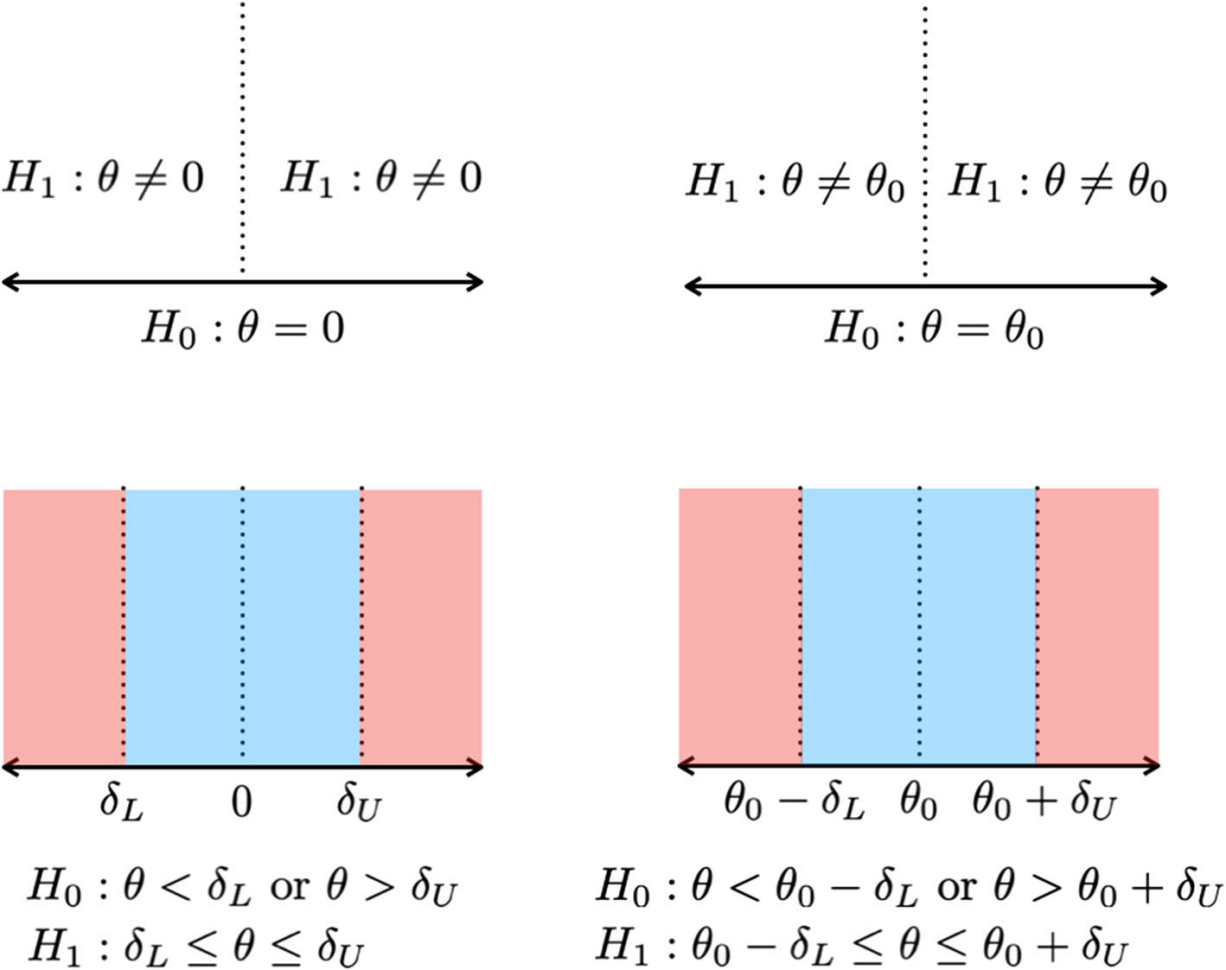
Differences between precise frequentist hypothesis testing and equivalence testing: Standard NHST for a sharp point null hypothesis *H*_0_:*θ*=0 against its alternative *H*_1_:*θ*≠0 (top left) or, in general, of *H*_0_:*θ*=*θ*_0_ against *H*_1_:*θ*≠*θ*_0_ (top right); TOST procedure for testing *H*_0_:*θ*<*δ*_*L*_ or*θ*>*δ*_*U*_ (shown in red) against *H*_1_:*δ*_*L*_≤*θ*≤*δ*_*U*_ (shown in blue) (bottom left) or *H*_0_:*θ*<*θ*_0_−*δ*_*L*_ or*θ*>*θ*_0_+*δ*_*U*_ (shown in red) against *H*_1_:*θ*_0_−*δ*_*L*_≤*θ*≤*θ*_0_+*δ*_*U*_ (shown in blue) (bottom right)

From the frequentist perspective, equivalence testing can be realized via two one-sided tests. Lakens et al. [38] gives a detailed account for this method which can be summarized as follows and was first proposed by Schuirmann [93], Anderson and Hauck [94, 95] and Rocke [96]: Instead of testing *H*_0_:*θ*=0 against *H*_1_:*θ*≠0 the frequentist equivalence test via the TOST procedure tests the hypotheses

6 $$\begin{array}{*{20}l} H_{0}:\theta < \delta_{L} \text{ or}\ \theta > \delta_{U} \text{ versus}\ H_{1}:\delta_{L} \leq \theta \leq \delta_{U} \end{array} $$

where *δ*_*L*_,*δ*_*U*_ are the lower and upper equivalence bounds. The situation and the contrast to precise hypothesis testing is illustrated in Fig. 12. The top right situation shows precise point null hypothesis testing of *H*_0_:*θ*=0 against *H*_1_:*θ*≠0. The top left situation is a special case for *θ*_0_=0, often termed the “no effect” hypothesis in practice. The bottom left situation shows equivalence testing, which tests *H*_0_:*θ*<*δ*_*L*_ or *θ*>*δ*_*U*_ (shown in red) against *H*_1_:*δ*_*L*_≤*θ*≤*δ*_*U*_ (shown in blue). This amounts to an equivalence hypothesis around the value *θ*_0_=0, and the bottom right situation is the general case where the equivalence bounds *δ*_*U*_,*δ*_*L*_ are placed around an arbitrary value *θ*_0_, not necessarily *θ*_0_=0. The name two one-sided tests stems from the fact that (6) can be implemented by first testing

7 $$\begin{array}{*{20}l} H_{01}:\theta < \delta_{L} \text{ versus}\ H_{11}:\theta \geq \delta_{L} \end{array} $$

When the result is statistically significant (e.g. for level *α*=0.05), one can then reject *H*_0_:*δ*<*δ*_*L*_. Second, one tests

8 $$\begin{array}{*{20}l} H_{02}:\theta > \delta_{U} \text{ versus}\ H_{12}: \theta \leq \delta_{U} \end{array} $$

If this second test turns out statistically significant, too (e.g. also for *α*=0.05), one can conclude that *δ*_*L*_≤*θ*≤*δ*_*U*_. In total, one can thus reject *H*_0_:*θ*<*δ*_*L*_ or *θ*>*δ*_*U*_ in (6). The combination of two one-sided hypothesis tests allows to establish an equivalence test. For example, choosing *δ*_*L*_=−0.2 and *δ*_*U*_=0.2 results in the equivalence test which tests if the parameter *θ* is smaller than −0.2 or larger than 0.2 versus the alternative that the parameter is inside [−0.2,0.2]. For extensions and modifications of the TOST procedure see Anderson & Hauck [94], Berger & Hsu [97], Schuirmann [70, 93], Meyners [98], Chow & Liu [99], and Wellek [100].

Note that the null and alternative hypothesis are reversed compared to Bayesian equivalence testing. However, in contrast to precise frequentist hypothesis testing, this is due the inability of frequentist methods to directly accept a hypothesis: Here, the hypothesis of equivalence is formulated as the alternative *H*_1_:*δ*_*L*_≤*θ*≤*δ*_*U*_, which can only be accepted by rejecting *H*_0_:*θ*<*δ*_*L*_ or *θ*>*δ*_*U*_, because concluding bioequivalence when it does not hold has serious consequences for the health of the public. Thus, we want to make sure that the probability of committing this error (type I error) is controlled. This is the reason that the formulation (6) is used in practice, and recommended by the US FDA and other regulatory agencies. In contrast to precise frequentist hypothesis testing, one can easily switch the null and the alternative in Eq. (6), and derive a corresponding test when considering frequentist equivalence testing. Details are also provided by Blackwelder [101].

### How to obtain the OH Bayes factor in the model of Morey and Rouder

To obtain the OH Bayes factor, Morey and Rouder [41] make use of the transitivity of Bayes factors, which allows to obtain a Bayes factor *BF*_13_ based on the Bayes factors *BF*_12_ and *BF*_23_ as follows:

$$\begin{array}{*{20}l} BF_{13}=BF_{12}BF_{23}=\frac{p(x|H_{1})}{p(x|H_{2})}\frac{p(x|H_{2})}{p(x|H_{3})}=\frac{p(x|H_{1})}{p(x|H_{3})} \end{array} $$

Denoting the Bayes factor of *H*_0_:*δ*=0 against *H*_1_:*δ*∼*C*(0,*r*_*i*_) as *BF*_01_(*r*_*i*_),*i*=0,1, the OH Bayes factor of the null $H_{0}^{\text {OH}}$ vs. the alternative $H_{1}^{\text {OH}}$ is then obtained via transitivity as

$$\begin{array}{*{20}l} BF_{01}^{\text{OH}}&=BF_{01}(r_{1})/BF_{01}(r_{0})\\ &=\frac{p(x|H_{0})}{p\left(x|H_{1}^{\text{OH}}\right)}/\frac{p(x|H_{0})}{p\left(x|H_{0}^{\text{OH}}\right)}=\frac{p\left(x|H_{0}^{\text{OH}}\right)}{p\left(x|H_{1}^{\text{OH}}\right)} \end{array} $$

Notice that obtaining the Bayes factors *BF*_01_(*r*_*i*_) in the JZS model which uses nil hypotheses for *i*=0,1 is straightforward via analytic formulas, compare Rouder et al. [28].

#### The hybrid model of Morey and Rouder

Morey and Rouder [41] even proposed a third model, the so-called hybrid model. The difference to the previous models in the hybrid model is that nil hypotheses like *H*_0_:*δ*=0 are now allowed to occur. Simultaneously, a small range of parameter values around the nil value *δ*=0 should be interpreted as zero again. The hybrid model consists of a two-component mixture given as follows:

$$\begin{array}{*{20}l} &H_{0}:\delta \sim \pi_{0} \cdot 1_{0} + \pi_{1} \cdot t_{\nu_{0}} \text{ for}\ \delta \in (-c,c)\\ &H_{1}:\delta \sim t_{\nu_{0}} \text{ for}\ \delta \notin (-c,c) \end{array} $$

That is, under the null in the hybrid model, the prior probability for a precise nil effect *δ*=0 is *π*_0_. The prior probability for a null effect as specified in the NOH model, that is, $\delta \sim t_{\nu _{0}}$ for *δ*∈(−*c*,*c*) is *π*_1_=1−*π*_0_. The corresponding Bayes factor $BF_{01}^{\text {hybrid}}$ is given as

$$\begin{array}{*{20}l} BF_{01}^{\text{hybrid}}=\frac{\pi_{0} p\left(x|H_{0}^{\text{JZS}}\right)+(1-\pi_{0})p\left({\vphantom{x|H_{0}^{\text{JZS}}}}x|H_{0}^{\text{NOH}}\right)}{p\left({\vphantom{x|H_{0}^{\text{JZS}}}}x|H_{1}^{\text{NOH}}\right)} \end{array} $$

Of course, in the hybrid model, the prior probability *π*_0_ needs to be chosen. Interestingly, for *c*→0, the hybrid model recovers the JZS model. With *π*_0_→0, the model recovers the NOH model.

However, while the hybrid model seems appealing at first glance, Morey et al. [41] themselves note: “For researchers who believe that nil hypotheses are impossible a priori, or who are uninterested in the nil, *π*_0_=0 is a reasonable value.”Morey et al. ([41], p. 26)

Then, the NOH model is recovered. In most biomedical research, the presence of exact nil effects is highly questionable [35], so that the suitability of the hybrid model for biomedical research settings seems questionable, too. What is more, the selection of the parameter *π*_0_ presents an additional challenge compared to the NOH or OH model: The parameter *π*_0_ resembles the a priori assumption about the proportion of exact nil effects in the research domain. First, this proportion cannot be estimated reliably even under sufficient domain-specific knowledge. Second, it is often unrealistic to assume any value *π*_0_>0 in medicine, psychology or the cognitive sciences. As a consequence, in this paper the hybrid model is excluded from the analysis and only the OH and NOH models proposed by Morey et al. [41] are studied, as they are more realistic for biomedical research.

#### Details on the Bayes factor of Van Ravenzwaaij et al.

Instead of choosing the precise null hypothesis *H*_0_:*δ*=0 and alternative *H*_1_:*δ*≠0, Van Ravenzwaaij et al. [44] allow for equivalence testing by considering *H*_0_:*δ*∼*C*(0,*r*_0_),*δ*∈(−*c*,*c*) and *H*_1_:*δ*∼*C*(0,*r*_1_),*δ*∉(−*c*,*c*). Still, these hypotheses are identical to the hypotheses considered in the non-overlapping hypotheses model of Morey and Rouder [41], and the Bayes factor is inspired by the solution of Gronau et al. [31] (compare Corollary A.2.3 in Gronau *et al* [31]) and is computed via the same numerical integration routine as

$$\begin{array}{*{20}l} BF_{01}&=\frac{\mathbb{P}(H_{0}|x)}{\mathbb{P}(H_{1}|x)}/\frac{\mathbb{P}(H_{0})}{\mathbb{P}(H_{1})}=\frac{\mathbb{P}(H_{0}|x)}{\mathbb{P}(H_{0})}/\frac{\mathbb{P}(H_{1}|x)}{\mathbb{P}(H_{1})}\\ &=\frac{\frac{\int_{-c}^{c} T_{\nu}(t|\sqrt{n}_{\delta} \delta)C(\mu_{\delta},\gamma_{\delta})d\delta}{\int_{-c}^{c} C(\mu_{\delta},\gamma_{\delta})d\delta}}{\frac{\int_{\Theta_{\delta}^{c}} T_{\nu}(t|\sqrt{n}_{\delta} \delta)C(\mu_{\delta},\gamma_{\delta})d\delta}{\int_{\Theta_{\delta}^{c}}C(\mu_{\delta},\gamma_{\delta})d\delta}} \end{array} $$

where $\Theta _{\delta }^{c}=(-\infty,-c]\cup [c,\infty)$. Van Raavenzwaaij selected *μ*_*δ*_=0 and $\gamma _{\delta }=1/\sqrt {2}$, which is the recommended standard setting according to Rouder et al. [28]. However, a wide *C*(0,1) prior would be an alternative in the setting of the two-sample t-test to prevent cherry-picking and retain objectivity, compare Kelter [40].

## Data Availability

The datasets generated and/or analysed during the current study as well as a full replication script to reproduce all results are available in the Open Science Framework (OSF) repository, https://osf.io/2cs75/.

## Abbreviations

NHST: Null hypothesis significance testing
BF: Bayes factor
ROPE: Region of practical equivalence

## Footnotes

1 See Lehmann [15] for a balanced perspective which focusses on the appropriate frame of reference of the statistical inference.

2 In this paper Bayesian equivalence tests are investigated. Frequentist equivalence tests, superiority tests or non-inferiority tests are not studied, although the Bayesian versions of the latter two can be identified as slight modifications of Bayesian equivalence tests, which is clarified in the main text later.

3 However, the Cauchy distribution has fat tails so it could also be reasonable to use distributions with lighter tails as an alternative (for example, a normal distribution).

4 Van Ravenzwaaij et al. [44] also present examples of how to apply the Bayes factor for non-inferiority and superiority testing, for details see the original paper.

5 Results show that this is not the case.

6 Note that in this case, the support interval should not be given a Bayes factor interpretation: The interval includes parameter values which have been corroborated by the data, that is *p*(*θ*|*x*)/*p*(*θ*)>*k*. While the Savage-Dickey Bayes factor representation allows to interpret values inside the support interval as yielding *B**F*_01_>*k*, to reject the null based on Bayes factors one would logically require parameter values which yield a *B**F*_10_>*k*. Thus, in general, the support interval draws its legitimation from including values which have been corroborated by the data, and not by the fact that sometimes a Bayes factor interpretation can be given to them.

7 An exception is given by the OH model of Morey et al. [41], where only the widths *r*_0_ and *r*_1_ need to be specified. However, *r*_0_ can be interpreted as the width of the equivalence region in the OH model.

8 In the frequentist paradigm a widespread equivalence testing procedure is the two one-sided tests (TOST) procedure described in Lakens et al. [38], see Appendix A.

9 Similar proposals for default ROPEs as *β*=0.05 for regression coefficients have been made for logistic and linear regression models. For a mathematical derivation see Kruschke ([36], p. 277).

10 Lakens et al. [38] underline that this is the weakest possible justification.

11 Other options next to the JZS model of Rouder et al. [28] which is used to compute the NOH model of Morey and Rouder [41] would be the Bayesian t-test models of Kruschke [58] or Kelter [32, 71].

12 The Bayes factor of Van Ravenzwaaij et al. [44] is not reported here because it is identical to the NOH model of Morey et al. [41], but it can be computed using the baymedr R package [45], see the provided replication script.

13 An exception is the OH model of Morey et al. [41], in which the associated Bayes factor is not consistent as discussed above.

14 The NOH Bayes factor $BF_{01}^{NOH}$ for *R*=[−0.05,0.05] yields 9.27 under the wide *C*(0,1) prior in this case, indicating also moderate evidence for the interval null hypothesis, compare Jeffreys [60].
